# Exosomes Released by Corneal Stromal Cells Show Molecular Alterations in Keratoconus Patients and Induce Different Cellular Behavior

**DOI:** 10.3390/biomedicines10102348

**Published:** 2022-09-21

**Authors:** Víctor Lozano, Carla Martín, Noelia Blanco, Ignacio Alcalde, Luis Fernandez-Vega Cueto, Jesús Merayo-Lloves, Luis M. Quirós

**Affiliations:** 1 Department of Functional Biology, University of Oviedo, 33006 Oviedo, Spain; lozanoiturbevict.fuo@uniovi.es (V.L.); cmartincueto@gmail.com (C.M.); uo257460@uniovi.es (N.B.); 2 Instituto Universitario Fernández-Vega, Fundación de Investigación Oftalmológica, University of Oviedo, 33012 Oviedo, Spain; nacho.alcalde@fio.as; 3 Instituto de Investigación Sanitaria del Principado de Asturias (ISPA), 33011 Oviedo, Spain

**Keywords:** exosomes, keratoconus, corneal stroma, proteomics, miRNA

## Abstract

Exosomes have been related to various disorders, but their study in relation to ocular pathologies has been limited. In this article, we analyze exosomes produced by corneal stromal cells from healthy individuals and from patients with keratoconus. The proteomic study allowed for the identification of 14 new proteins with altered expression, related to molecules previously associated with the pathology. miRNA analysis detected 16 altered species, including miR-184, responsible for familial severe keratoconus. The prediction of its potential biological targets identified 1121 genes, including some related to this pathology. Exosomes produced by keratoconic cells induced a marked increase in the migration of stromal cells and corneal epithelium, while those produced by healthy cells had no effect on stromal cells. Both types of nanovesicles reduced the proliferation of stromal and corneal cells, but those produced by healthy cells had less effect. Exosomes produced by healthy cells had concentration-dependent effects on the transcription of genes encoding proteoglycans by keratoconus cells, with a relative normalization observed at concentrations of 240 µg/mL. These results show the alteration of stromal exosomes in keratoconus and suggest an influence on the development of the pathology, although the use of healthy exosomes could also have therapeutic potential.

## 1. Introduction

Keratoconus (KC) is the most common type of degenerative eye disease that involves deformation of the cornea. There is a progressive reduction in corneal thickness, which causes ectasia and an increase in superficial curvature. As a consequence, the cornea acquires a typical conical shape, which can lead to significant visual impairment [1,2].

KC is a complex multifactorial disorder whose molecular and pathological mechanisms are not well understood. On the one hand, although an inconclusive genetic link has been established, between 8 and 10% of cases have a hereditary component, and a possible relationship with certain genes, such as LOX, VSX1, SOD1 and mutations in MIR184 have been described [3,4,5,6,7]. However, various environmental factors also seem to be related to the pathology, acting as stimuli capable of triggering the aberrant tissue response regardless of the existence or not of a genetic component. These factors include wearing contact lenses, eye rubbing and ultraviolet light exposure [4,8,9].

KC is a pathology that can appear isolated or associated with others of a different nature. Among the disorders with which it has been associated include ocular diseases such as Leber congenital amaurosis, vernal disease and retinitis pigmentosa [4,10], allergic immune disorders and autoimmune diseases [11] and, in particular, connective tissue diseases such as Ehlers-Danlos syndrome, mitral valve prolapse, osteogenesis imperfecta, joint hypermobility disease, Marfan syndrome and pseudoxanthoma elasticum [4,8,12,13,14]

At the histopathological level, KC is characterized by the presence of a degraded stroma, and the depletion and abnormal behavior of keratocytes, which appear to be poorly differentiated, indicating loss of their quiescent phenotype [1,3]. Epithelial cells also appear degenerated, showing increased cell thinning, a reduced cell density and various changes in basal epithelial cells [15,16]. Other corneal layers are also affected, with breaks in the Bowman’s layer and the occasional protrusion of epithelial cells or keratocytes having been observed [8].

Various studies have addressed the molecular alterations on which the histological and morphological changes associated with the pathology may be based. Some proteomic studies carried out on corneal epithelium in KC have shown alterations related to the cytoskeleton and that they are involved in cell-cell or cell-basement membrane associations [17,18]. In studies of the KC stroma, alterations observed include changes in the level of a number of proteins similar to those that have been observed to be linked to wound healing and the loss of the quiescent phenotype of keratocytes [19,20]. Stromal alterations also include a global decrease in different types of collagens, consistent with clinical observations of the thinning of the corneal thickness [19,20] as well as in various proteoglycans (PGs) associated with the extracellular matrix (ECM) and some cell surface PGs. Changes in the sulfation patterns of the glycosaminoglycan chains (GAGs) associated with these molecules have also been observed [1,21]. In addition, there is evidence of an increase in the expression of proteolytic enzymes associated with the development of KC, as well as in heparanase, an endo-β-D-glucuronidase that cleaves specific linkages in the structure of the GAG heparan sulfate [1,22].

PGs are complex molecules involved in the regulation of numerous essential processes of cell physiology [23]. Mainly through their GAG chains, they are capable of specifically binding various ligands involved in cell signaling, such as cytokines, chemokines and growth factors, controlling their function and concentration. Among these ligands, there are several that show an aberrant expression in KC [24,25]. These molecules also play essential roles in relation to the biogenesis, secretion and composition of exosomes, as well as in their internalization by the recipient cell and their functional activity within it [26], where heparanase plays a part [27].

Exosomes are nanovesicles released into the extracellular environment by almost all types of cells, under both healthy and pathological conditions. They are characterized fundamentally by their size, between 30 and 150 nm in diameter, as well as by the molecules of which they are comprised, especially proteins and various species of RNAs [28,29]. Exosomes play an essential role in intercellular communication, for which their composition is essential. When exosomes come into contact with receptor cells, they can undergo immediate stimulation through cell signaling mediated by exosomal proteins, or else their cellular behavior may be modified by the action of proteins, microRNAs or mRNAs present in the vesicles [30,31] As a result, exosomes participate in the control of important biological functions, including immunomodulation, regeneration, differentiation, inflammation, neovascularization, and cellular waste removal [32,33].

Exosomes have been related to many pathologies, including amyloid protein transfer, the promotion of tumor expansion, involvement in heart disease, and cystic fibrosis and the transmission of molecules related to pathogens or the stimulation of the immune response [34]. In relation to ocular pathologies, the knowledge of the role of exosomes is, however, very limited. Some studies have linked them with the dry-eye symptoms associated with Sjögrens syndrome, with the rejection of corneal grafts, with uveitis, glaucoma and age-related macular degeneration [33]. However, there are no studies that address the study of exosomes in relation to KC.

In this article, we isolate exosomes produced by corneal stromal cells from healthy donors and from patients with KC. Two of the most important molecular components of these nanovesicles, proteins and miRNAs, were analyzed, with 14 significant differences being found in the former and 16 in the latter. None of the proteins with altered expression have previously been reported as related to this pathology, although a bioinformatic analysis revealed the existence of functional connections between these molecules. On the other hand, among the targets of the miRNAs with altered expression, genes previously related to the pathology were identified. The addition of exosomes to corneal stromal cell cultures induced changes in their migration and proliferation depending on the origin, healthy or pathological, of the nanovesicles. Stromal exosomes also induced behavioral changes in corneal epithelial cells. In addition, the addition of exosomes from healthy cells to keratoconic cells modified the alterations in the transcription of genes encoding heparan sulfate proteoglycans (HSPGs) and small leucine-rich proteoglycans (SLRPs) in a concentration-dependent manner. Taken together, these results point to a role for exosomes in the development of this pathology, as well as their potential therapeutic use.

## 2. Materials and Methods

### 2.1. Tissue Samples

All of the corneas used in this study were obtained from the Instituto Oftalmológico Fernández-Vega and from the Central University Hospital of Asturias (both in Asturias, Spain). Informed oral and written consent of the patients or their relatives was obtained under a protocol approved by the approved by the Ethics Committee of Principado de Asturias (Protocol Code: 2020.50. 23 March 2020), in accordance with the guidelines of the Tenets of the Declaration of Helsinki.

### 2.2. Isolation and Culture of Corneal Stromal Cells from Healthy Human Donor Corneas and Keratoconic Corneas

Human central corneal tissue was obtained from 3 cadaver donors and from 3 penetrating keratoplasty interventions on patients suffering from KC. All of the cases were white males, aged 42, 58, and 67 years (healthy donors) and 45, 46, and 64 years (keratoconus patients). Healthy donor tissues were screened and tested negative for HIV, hepatitis B and C virus and syphilis, and were not usable for human corneal transplantation.

The epithelium was removed with ethanol (70%, 30 s) and a spatula, and the endothelium by Descemet membrane endothelial keratoplasty, after which the absence of epithelial and endothelial cells was assessed by microscopy. Corneal stromal cells were obtained by digesting 2-mm diameter pieces from the central cornea in 0.25% trypsin/ethylenediaminetetraacetic acid solution for 30 min at 37 °C. After centrifugation, the supernatant was discarded and the pellet resuspended in a DMEM + F12 culture medium containing non-essential amino acids, RPMI 1640 Vitamin Solution 100×, 1% antibiotics (penicillin/streptomycin) and 10% fetal bovine serum (Gibco, Waltham, MA, USA). When cultures reached 80% confluence, they were replated at a density of 2 × 10^5^ cells/mL in 75-cm^2^ flasks. As a control to evaluate whether the cells maintained a stable phenotype, we performed alpha-smooth muscle actin (αSMA) immunostaining as previously described [35]. Briefly, cells were incubated overnight with a rabbit polyclonal anti αSMA antibody (Abcam, Cambridge, UK), followed by a 2 h incubation with a complementary fluorescent antibody against rabbit IgG (AlexaFluor 488; Invitrogen, Eugene, OR, USA). Nuclei were counterstained with DAPI (4′,6-diamidino-2-phenylindole dihydrochloride; Invitrogen, Waltham, MA, USA). αSMA staining remained negative until the fifth passage, suggesting that the stromal cells did not readily differentiate into myofibroblasts (Supplementary Figure S1). As a result, only cultures in passages 1–5 were used. In all of the experiments carried out in this work, the various cell lines were analyzed separately.

### 2.3. Corneal Epithelial Cell Lines

For the studies of the effect of the addition of exosomes on corneal epithelial cells, the HCE-2 (50.B1) ATCC CRL-11135 line was used. This corneal epithelial cell line was grown in Dulbecco’s Modified Eagle’s minimal essential medium (DMEM). The culture broth was supplemented with 10% (*w*/*v*) fetal bovine serum, 1.5 units/mL of insulin, 10 ng/mL of epidermal growth factor (EGF), and with penicillin G/streptomycin (5000 IU/mL, 5000 µg/mL). Cultures were incubated at 37 °C in a 5% (*v*/*v*) CO_2_ atmosphere.

### 2.4. Exosome Purification

For the purification of the exosomes, plates containing cells grown to a confluence of between 50 and 60% were used, the culture medium being eliminated and replaced by an equivalent volume where the FBS was replaced by an exosome-depleted FBS (Gibco). After 72 h of incubation, the culture medium was collected and the exosomes were obtained by two different procedures depending on how they were to be used in subsequent experiments. For the characterization of the nanovesicles by nanoparticle tracking analysis (NTA) or electron microscopy, as well as for their subsequent addition to cell cultures, the exosomes were purified using the “total exosome isolation from cell culture media” kit (Invitrogen, USA), following the manufacturer’s instructions. Briefly, the conditioned medium was centrifuged at 2000× *g* for 30 min to remove cells and cell debris, and 0.5 volumes of “Total Exosome Isolation” reagent were added to the supernatant. After homogenization, the mixture was incubated overnight at 4 °C. After subsequent centrifugation at 10,000× *g* for 1 h at 4 °C, the pellet, containing the exosomes, was resuspended in 200 μL of PBS.

The exosomes destined for peptide analysis by LC-MS/MS were purified using Exo-spinTM mini columns (CELL guidance systems, Cambridge, UK) following the manufacturer’s specifications, with some modifications. After centrifuging the conditioned medium at 300× *g* for 10 min to remove cells and cell debris, the precipitation step prior to its addition to the purification column was replaced by ultracentrifugation at 110,000× *g* for 2.5 h. This modification was made to prevent the precipitant from interfering with the later mass spectrometric analysis.

Once purified, the exosomes were quantified by determining the protein concentration with the Pierce BCA Protein Assay Kit (Thermo Scientific, Waltham, MA, USA), following the manufacturer’s instructions.

### 2.5. NanoSight Particle Size Analysis Using Dynamic Light Scattering

A 1:100 dilution of the exosomes was prepared in PBS, from which 1:10 and 1:100 dilutions were subsequently obtained using the same buffer. The dilutions were injected into a Malvern Panalytical NanoSight LM10 instrument, starting with the one with the lowest concentration, until the one that provided a number of particles between 21–100 in the observed field was identified. The analysis was carried out with a 405 nm laser, and the light scattered by the particles was analyzed using the NTA analysis software 3.1 (Nanosight, Westborough, MA, USA) to determine the size of the particles and their concentration.

### 2.6. Transmission Electron Microscopy (TEM)

A total of 20 microliters of the purified exosomes were adsorbed onto a carbon grid for 2 min. After drying off the excess sample, it was stained by depositing 50 microliters of 2% phosphotungstic acid on the grid for 1 min and allowing the sample to dry for 10 min. The samples were examined in a JEOL JEM 2000 EX II electron microscope at 120 Kv.

### 2.7. In-Gel Protein Digestion for Proteomic Analysis

Exosomes were diluted with loading sample buffer and applied to a conventional SDS-PAGE gel. The run was stopped as soon as the front had penetrated 1 cm into the resolving gel, after which the unseparated protein bands were visualised by Coomassie staining, excised, cut into cubes (1 mm^2^), deposited in 96-well plates and processed automatically in a Proteineer DP (Bruker Daltonics, Bremen, Germany). The digestion protocol used was based on Schevchenko et al. [36] with minor variations: gel plugs were washed, firstly with 50 mM ammonium bicarbonate and secondly with ACN, prior to reduction with 10 mM DTT in 25 mM ammonium bicarbonate solution, and alkylation was carried out with 55 mM IAA in 50 mM ammonium bicarbonate solution. Gel pieces were then rinsed, firstly with 50 mM ammonium bicarbonate and secondly with ACN, and then were dried under a stream of nitrogen. Proteomics grade trypsin (Sigma Aldrich, St Louis, MO, USA) at a final concentration of 16 ng/μL in 25% ACN/50 mM ammonium bicarbonate solution was added and the digestion took place at 37 °C for 4 h. The reaction was stopped by adding 50%ACN/0.5%TFA for peptide extraction. The tryptic eluted peptides were dried by speed-vacuum centrifugation.

### 2.8. Liquid Chromatography and Mass Spectrometer Analysis (LC-ESI-MS/MS)

A 1 µg aliquot of each digested sample was subjected to 1D-nano LC ESI-MSMS analysis using a nano liquid chromatography system (Eksigent Technologies nanoLC Ultra 1D plus, SCIEX, Foster City, CA) coupled to high-speed Triple TOF 5600 mass spectrometer (SCIEX) with a Nanospray III source. The analytical column used was a silica-based reversed phase Acquity UPLC^®^ M-Class Peptide BEH C18 Column, 75 µm × 150 mm, 1.7 µm particle size and 130 Å pore size (Waters, Milford, MA, USA). The trap column was a C18 Acclaim PepMapTM 100 (Thermo Scientific, Waltham, MA, USA) column of 100 µm × 2 cm, 5 µm particle diameter, 100 Å pore size, on-line switched with the analytical column. The loading pump delivered a solution of 0.1% formic acid in water at 2 µL/min. The nano-pump provided a flowrate of 250 nl/min and was operated under gradient elution conditions. Peptides were separated using a 150-min gradient ranging from 2% to 90% mobile phase B (mobile phase A: 2% acetonitrile, 0.1% formic acid; mobile phase B: 100% acetonitrile, 0.1% formic acid). The injection volume was 5 µL.

Data acquisition was performed with a TripleTOF 5600 System (SCIEX), using an ionspray voltage floating (ISVF) 2300 V, curtain gas (CUR) 35, interface heater temperature (IHT) 150, ion source gas 1 (GS1) 25, declustering potential (DP) 100 V. All data was analyzedd using information-dependent acquisition (IDA) mode with Analyst TF 1.7 software (SCIEX). For IDA parameters, a 0.25 s MS survey scan in the mass range of 350–1250 Da were followed by 35 MS/MS scans of 100 ms in the mass range of 100–1800 (total cycle time: 4 s). Switching criteria were set to ions greater than mass to charge ratio (*m*/*z*) 350 and smaller than *m*/*z* 1250 with charge state of 2–5 and an abundance threshold of more than 90 counts (cps). Former target ions were excluded for 15 s. IDA rolling collision energy (CE) parameters script was used for automatically controlling the CE.

### 2.9. Proteomics Data Analysis and Sequence Search

The mass spectrometry data obtained were processed using PeakView v2.2 Software (SCIEX) and exported as mgf files, which were searched using Mascot Server v2.6.0 (Matrix Science, London, UK) against *Homo sapiens* protein database from Uniprot (last update: 20170331, 141.978 sequences), together with commonly occurring contaminants.

Search parameters were set as follows: enzyme, trypsin; allowed missed cleavages, 2; carbamidomethyl (C) as fixed modification and acetyl (Protein N-term), pyrrolidone from E, pyrrolidone from Q and Oxidation (M) as variable modifications. Peptide mass tolerance was set to ±25 ppm for precursors and 0.05 Da for fragment masses. The confidence interval for protein identification was set to ≥95% (*p* < 0.05) and only peptides with an individual ion score above the 1% false discovery rates (FDR) at spectra level were considered correctly identified.

### 2.10. Differential Protein Expression and Bioinformatics Analysis

Among the proteins identified, those that presented a more consistent expression were selected; that is, those detected in at least all samples of the same type (healthy or KC). The exponentially modified protein abundance index (emPAI) values were normalized [37] and statistically analyzed using the nonparametric Mann-Whitney U test. Those proteins for which the analysis was statistically significant (*p* <0.05), and which presented cut-off values of ≥2-fold (up-regulation) or ≤0.5-fold (down-regulation) between samples, were accepted as differentially expressed.

The functional classification of proteins according to family, molecular function, biological process and pathway was carried out using the PANTHER (protein analysis through evolutionary relationships) classification system (http://pantherdb.org/; accessed on 28 July 2021) [38]. Protein-protein interaction networks were generated using STRING database program (https://string-db.org/; accessed on 21 July 2021) [39].

### 2.11. Small RNA Next Generation Sequencing (NGS)

Sample preparation: RNA was isolated from 4500 μL using the exoRNeasy Midi Kit S(Qiagen, Hilden, Germany) according to the manufacturer’s instructions with an elution volume of 14 μL.

Library preparation and sequencing was carried out using the QIAseq miRNA Library Kit (Qiagen). A total of 5μL total RNA was converted into miRNA NGS libraries. Adapters containing UMIs were ligated to the RNA. Then, RNA was converted to cDNA. The cDNA was amplified using PCR (22 cycles) and during the PCR indices were added. After PCR the samples were purified. Library preparation QC was performed using Bioanalyzer 2100 (Agilent, Santa Clara, CA, USA).

Based on quality of the inserts and the concentration measurements, the libraries were pooled in equimolar ratios. The library pools were quantified using qPCR and then sequenced on a NextSeq550/500 sequencing instrument following the manufacturer’s instructions. Raw data was de-multiplexed and FASTQ files for each sample were generated using the bcl2fastq software (Illumina Inc, San Diego, CA, USA).

### 2.12. Differential miRNA Expression and Bioinformatics Analysis

Among the miRNAs identified, those that were most consistently expressed were selected, namely, those identified in at least all samples of the same type (healthy or KC). The values were normalized and statistically analyzed using the nonparametric Mann-Whitney U test. miRNAs where the analysis was statistically significant (*p* < 0.05), and which presented cut-off values of ≥2-fold (up-regulation) or ≤0.5-fold (down-regulation) between samples were considered to be differentially expressed.

To identify the proteins regulated by the significantly altered miRNAs, biological targets of miRNAs were predicted by searching for the presence of conserved 8 mer and 7 mer sites that match the seed region of each miRNA, using TargetScanHuman 5.2 (http://www.targetscan.org/vert_50/; accessed on 24 November 2021) [40,41,42] and selecting the targets with scores higher than 80 out of 100. Subsequently, the results of the previous selection were analyzed using the online database for miRNA target prediction and functional annotations, miRDB (http://mirdb.org/; accessed on 25 November 2021) [43,44], selecting the matching results from both tools.

### 2.13. Cell Migration and Cell Proliferation Assays 

Cell migration and proliferation assays were performed using culture medium including exosome-depleted FBS. For the different experiments, exosomes were added at a concentration of 60 µg protein/mL. Cell migration assays were performed by in vitro scratch assay using 24-well plates as previously described [45], measuring the scratch closure in the case of epithelial cells, and counting the number of cells that appear in the scratch area when it was stromal cells. For cell proliferation assays, stromal cells were seeded in 96-well plates (Thermo Fisher) at a density of 1500 cells/well. The effect of the addition of exosomes on cell proliferation was evaluated by a colorimetric assay using MTT (Promega, Madison, WI, USA) following the manufacturer’s instructions. Briefly, 15 µL of the MTT solution was added to each well and incubated for 4 h at 37 °C. Then, 100 µL of the lysis solution was added and the mixture was incubated at 4 °C overnight. The absorbance at 570 nm in each set of samples was measured using a Biotek Powe Wave XS 96-well microtiter plate reader (Biotek, Winooski, VT, USA). Five repetitions were carried out for each of the treatments.

### 2.14. Total RNA Isolation and cDNA Synthesis

RNA isolation was conducted using the RNeasy kit (Qiagen), starting from tissue fragments of between 20 and 30 mg in weight, and proceeding as has been previously described [46].

For the synthesis of the cDNA, 2 μg of RNA were used, and the reactions performed using the High Capacity cDNA Transcription Kit (Applied Biosystems, Foster City, CA, USA). The procedure was carried out and the products cleaned and stored as has been previously described [46].

### 2.15. qRT-PCR Reactions

The primers located in the different exons were designed using the program Primer 3 (http://biotools.umassmed.edu/bioapps/primer3_www.cgi; accessed on 5 January 2018), adjusting the amplicon size to between 70 and 150 base pairs, using a Tm of above 77 °C whenever possible. Primer sequences are presented in Supplementary Table S1.

Both the reactions and the analysis of the amplification products were carried out as has been described elsewhere [46]. Normalization of expression values was carried out using actin as a control gene. Statistical analysis of the data was carried out using the Mann–Whitney U test to compare the mean values, as described in a previous work [46].

## 3. Results

### 3.1. Characterization of Exosomes Derived from Corneal Stromal Cells

**Figure 1:**
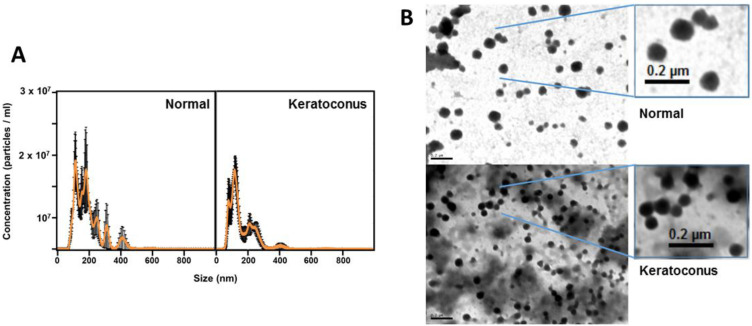
Characterization of normal- and KC corneal stromal cell derived extracellular vesicles. (**A**) Size distribution of vesicles determined by NanoSight LM10; left: normal stromal cells, right: keratoconic cells. (**B**) TEM images of exosomes from normal stromal cells (upper panel) and KC stromal cells (bottom panel).

The size of the extracellular vesicles purified from the conditioned medium of the corneal stromal cells cell lines was ascertained by NanoSight particle size analysis. Samples from patients with KC showed a typical KC particle size distribution, averaging around 113 nm (Figure 1A). For their part, vesicles isolated from healthy stromal cells showed a main peak also located at 113 nm, but a second peak corresponding to particles whose mean size was located at 178 nm also appeared (Figure 1A). When the samples were analyzed by TEM, in both cases vesicles with relatively homogeneous sizes were observed and located within the limits established for exosomes (Figure 1B).

### 3.2. Proteomic Analysis of Normal Versus Keratoconus Stromal Cell Exosomes

LC-MS/MS-based proteomic analysis identified 314 different proteins in exosomes isolated from healthy stromal cell cultures and 301 in isolates from KC cells, of which 65 and 54, respectively, were present in all of the samples tested. A complete list of proteins is shown in Supplementary Table S1. We were able to determine that the proteins included commonly identified exosome proteins from other cell types, with 17 of the 25 most frequently identified proteins in exosomes (68%) and 31 if considering the 50 most frequent (62%) being detected (http://exocarta. org/exosome_markers_new; accessed on 17 June 2021) (Supplementary Table S2). Among the proteins identified, several of the 30 that are most abundant in the corneal stroma were found [47], including COL1A1, COL1A2, LUM, COL6A1, COL5A1, ENO1 and VIM, although many were not detectable, among them KERA, COL4A3, COL5A2, IGHG4, ALDH3A1, BGN, MAMDC2, PRELP and SERPINA3. Similarly, some of the adhesion and extracellular matrix proteins whose expression in the corneal stroma has been previously described were also detected [48], including proteins with abundant expression such as FN1 and COL5A1, but also others such as TIMP1, CD44, THBS1 and CTNNB1; however, many others were not detected, including molecules that occur very frequently in both the central and peripheral stroma such as COL12A1 and ITGA6.

**Figure 2:**
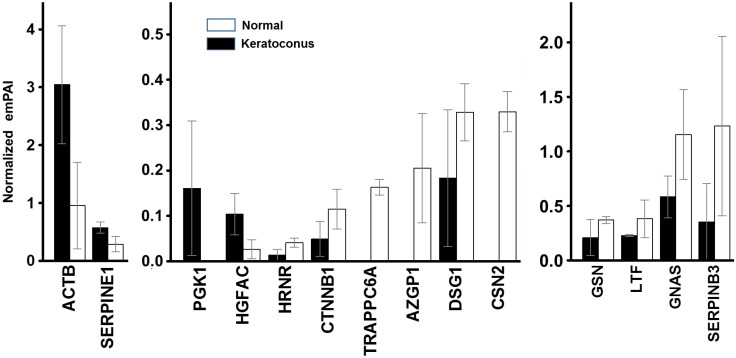
Proteins whose relative abundance levels in exosomes produced by stromal cells from healthy corneas and from KC show statistically significant differences (Mann-Whitney U test). The normalized emPAI values for healthy corneas (white bars) and KC (black bars) are shown. The spreads represent standard deviation.

Among the proteins identified in the samples, there were 79 that were consistently detectable in all samples of the same type, that is, in either healthy or KC samples, and these were selected for statistical analysis (Table 1). A total of 10 of these proteins showed statistically significant differences. Of these, 3 appeared over-expressed in the isolated exosomes of corneal stroma cells from patients with KC compared to healthy individuals (ACTB, SERPINE1, HGFAC), while 7 were under-expressed (HRNR, CTNNB1, DSG1, GSN, LTF, GNAS, SERPINB3). In addition, there were 3 proteins present only in healthy samples (TRAPPC6A, AZGP1, CSN2), while 1 was only detectable in KC (PGK1) (Figure 2).

**Figure 3:**
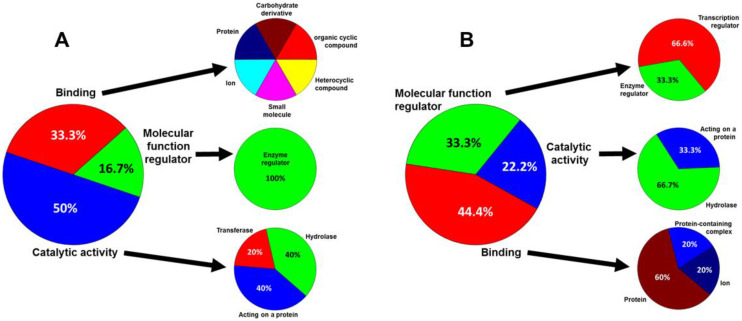
Identification of the molecular functions of the proteins whose relative abundance levels in exosomes produced by stromal cells from healthy corneas and from KC show statistically significant differences. (**A**) Proteins over-expressed in KC exosomes. (**B**) Proteins under-expressed in KC exosomes.

The identification of the molecular functions of the over-expressed proteins in KC using the PANTHER) classification system showed that they were mainly related to catalytic functions (50%) or related to binding (33.3%), and to a lesser extent as regulators of molecular functions (16.7%) (Figure 3). The detailed analysis of the proteins involved in catalysis showed that they were mainly constituted by hydrolases (40%) and by molecules that act on other proteins (40%) (Figure 3A). The proteins involved in binding act on different molecules in similar proportions, including proteins, ions, organic cyclic and heterocyclic compounds, carbohydrate derivatives and small molecules (Figure 3A). Finally, the minor proportion of molecular function regulators seem to act entirely as enzymatic regulators (Figure 3A).

The study of the molecular functions of the under-expressed proteins in KC gave different results to those that were over-expressed. While the proportion of proteins involved in binding was very similar (44.4%), in this case the vast majority were involved in interactions with proteins (60%) or protein-containing complex (20%) (Figure 3B). The percentage of proteins involved in catalysis was reduced by half (22.2%), with two two-thirds of them being hydrolases and the remaining third, molecules that act on proteins. Finally, the percentage of proteins with molecular regulatory functions was more than 2.3 times that of the over-expressed proteins, with most of them being involved in the regulation of transcription (Figure 3B).

**Figure 4:**
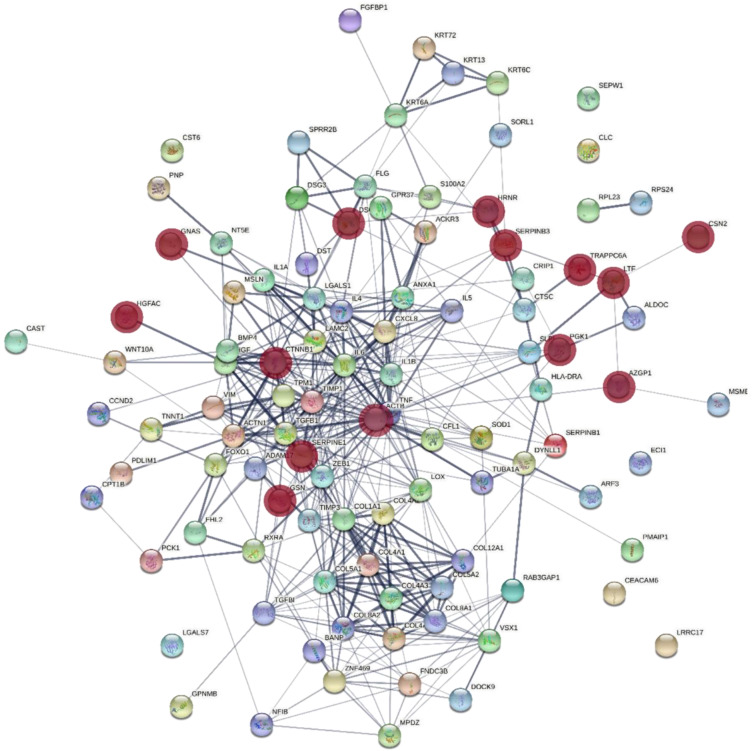
Functional association network of genes associated with central corneal thickness and KC. The STRING program was used to model the functional association of proteins whose expression is altered in exosomes of corneal stromal cells of patients with KC identified in this study (red nodes) with other genes related to the pathology in other studies cited in the bibliography.

The 14 proteins whose levels appeared altered in the exosomes produced by the corneal stromal cells of patients with KC did not coincide with the products of the genes that have been previously associated with the development of the pathology in the literature. To identify the existence of functional relationships between these molecules, we carried out a functional association network study using the STRING program, including 89 previously mentioned genes. The results are shown in Figure 4. We found that 4 of the proteins were associated with the core cluster, suggesting that they could likely play any role in relation to the pathology. Furthermore, the analysis of the molecular functions of the genes that make up this core cluster using the PANTHER program, related them preferentially to binding (41.2%) and molecular regulators (35.3%). The remaining molecules were located on the periphery, suggesting that they may be involved in some basic cell processes. In this sense, 7 of the proteins maintain a certain degree of relationship, and the analysis of their molecular functions using PANTHER related them preferentially to catalytic activities (45%).

### 3.3. miRNA Profile Analysis of Normal Versus Keratoconus Stromal Cell Exosomes

**Figure 5:**
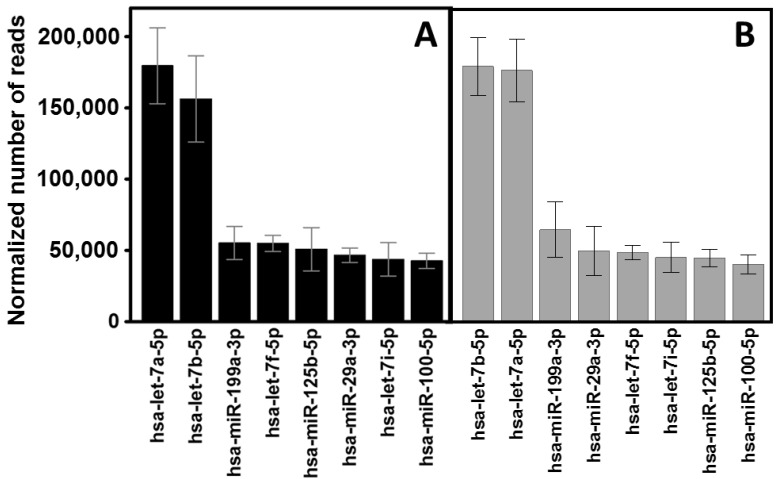
Expression of the 8 most abundant miRNAs in corneal stromal cell exosomes. (**A**) healthy cells. (**B**) KC cells. The spreads represent standard deviations.

Using NGS, the average sequence reads for normal and KC samples were 962,269 (SD 84,988) and 1046,592 (SD 122,020), respectively. After normalization, we were able to detect 800 different miRNAs, 692 of them present in exosomes isolated from healthy cells and 642 in pathological exomes. A complete list of miRNAs indicating the samples in which they were identified can be found in Supplementary Table S3. Among the 8 most abundant species, hsa-let-7a-5p and hsa-let-7b-5p stood out, exceeding 150,000 reads in both healthy cells and KC. The 6 next-most-abundant species were found at levels between 3 and 7 times lower (Figure 5). Overall, the exosome samples isolated from healthy stromal cells had a mean of 1451 readings, with a median of 12.4, while in KC the mean was 1564 and the median of 14.5 readings. The distribution of global abundances did not show any significant differences.

**Figure 6:**
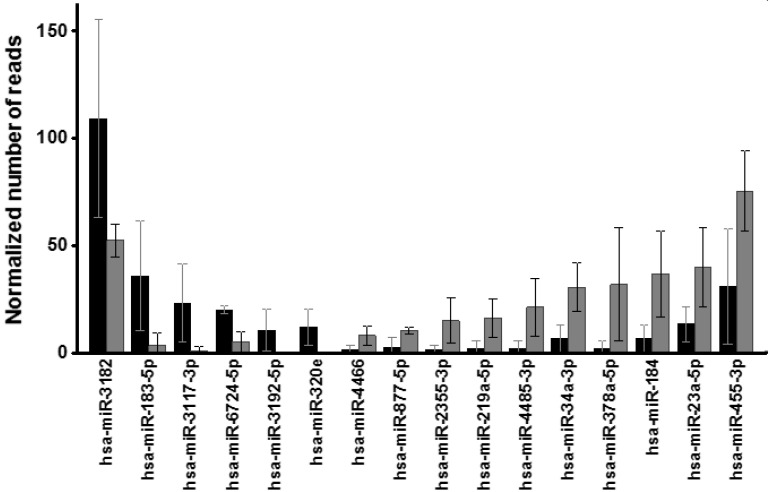
miRNAs whose relative abundance levels in exosomes produced by stromal cells from healthy corneas and from KC show statistically significant differences (Mann-Whitney U test). The normalized number of reads values for healthy corneas (black bars) and KC (gray bars) are shown. The spreads represent the standard deviation.

To carry out a differential expression analysis, the 344 miRNA species that appeared consistently in all samples of the same type were identified. In total, 286 different miRNAs were selected in the healthy samples and 301 in the KC ones. A list of these species along with the average number of normalized reads is shown in Supplementary Table S4. A total of 16 miRNAs showed statistically significant differences. Of these, 4 appeared over-expressed in exosomes isolated from corneal stromal cells from healthy patients (hsa-miR-3182, hsa-miR-183-5p, hsa-miR-3117-3p and hsa-miR-3192-5p), while 10 appeared over-expressed in KC exosomes (hsa-miR-4466, hsa-miR-877-5p, hsa-miR-2355-3p, hsa-miR-219a-5p, hsa-miR-4485-3p, hsa-miR-34a-3p, hsa-miR-378a-5p, hsa-miR-184, hsa-miR-23a-5p and hsa-miR-455-3p). Furthermore, 2 species were only detectable in healthy samples (hsa-miR-3192-5p and hsa-miR-320e). At least one of these miRNAs with altered expression, miR-184, has been previously associated with the development of KC [49]. These results are summarized in Figure 6.

The prediction of the biological targets of the altered miRNAs allowed for the identification of 1121 genes that are potentially regulated by 1 of these miRNAs, 126 by 2, 8 by 3 and 1 by 4 of the miRNA species with differential expression. The results are shown in Supplementary Table S5. Verification of whether any of the genes that encode the proteins whose expression is altered in KC exosomes could be a target of any of the miRNAs with differential expression showed the possible existence of post-transcriptional regulation of SERPINE1 by hsa-miR-2355-3p. When the analysis was extended to the multiple loci previously identified as being associated with KC, it was found that 7 different proteins could be regulated by some of the miRNAs that showed differential expression (Table 2).

### 3.4. Effect of Exosomes on the Expression of Genes of Proteoglycans and Glycosaminoglycans in Corneal Cells

Among the genes predicted as biological targets of miRNAs whose levels appear altered in the exosomes of keratoconic cells, there are several that encode PG core proteins, or are related to the glycosaminoglycan chains covalently linked to them (Supplementary Table S6). Given the sensitivity of these molecules to pathological conditions, and their fundamental role in the regulation of cell physiology and communication, as well as in the structuring of tissues and the extracellular matrix, we analyzed the influence that the biological information contained in exosomes could have on the transcription of some of these genes, including those responsible for the synthesis of HSPGs, and those encoding SLRPs.

**Figure 7:**
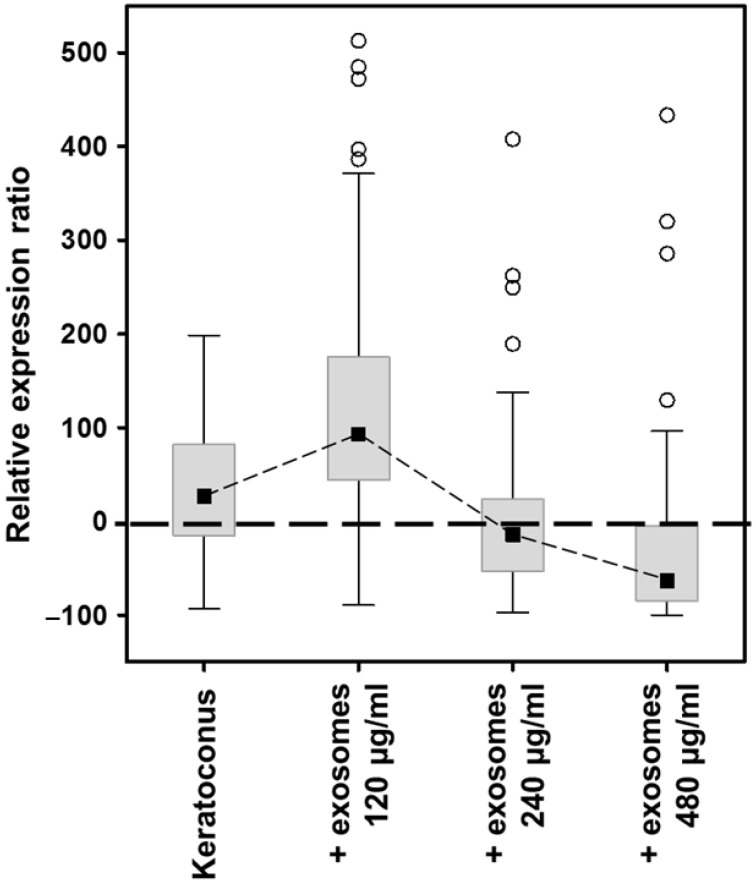
Alteration in the gene expression of KC cells induced by exosomes produced by healthy stromal cells. The relation of the relative expression of genes encoding HSPGs and SLRPs in corneal stromal cells of patients with KC cultured alone or in the presence of exosomes isolated from healthy cell cultures at concentrations of 120, 240 and 480 µg of protein/mL are shown. The expressions are referenced to the values of healthy cells, represented by the line at 0. The black squares represent the median of the values; the rectangles, the 25 to 75% range of values; the spreads the non-outlier range; and the circles the outliers.

Transcripts of 52 genes from these families were detected. Of them, 15 showed alterations in the KC samples. The addition of exosomes isolated from healthy stromal cells to KC cell cultures at a final concentration of 120 µg protein/mL increased the observed differences to 26, while the use of a concentration of 240 µg protein/mL reduced them to 11, although this was increased again to 26 when exosomes were added at 480 µg/mL (Supplementary Figure S2). As a whole, the median of the expression values of the 52 genes appeared increased by 27% in KC, a difference that increased to 92% with the addition of exosomes at a concentration of 120 µg of protein/mL, dropped to 15% below the control with the addition of 240 µg of protein/mL, and to 64% below the control figure when exosomes were added at a concentration of 480 µg of protein/ml (Figure 7).

### 3.5. Effect of Exosomes on Proliferation and Migration of Corneal Epithelial and Stromal Cells

**Figure 8:**
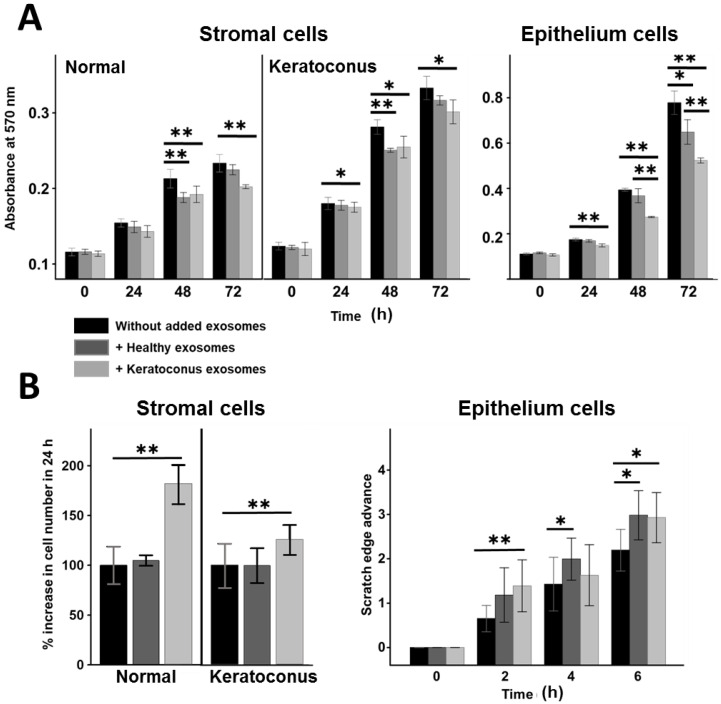
Influence of pathological exosomes on cell behavior compared to that produced by healthy cells. (**A**) Influence of exosomes on cell proliferation. (**B**) Influence of exosomes on cell migration. Values display the mean of 3 independent experiments, and the spreads represent standard deviation. Values that show significant differences (Student’s *t* test) are highlighted: *: *p* < 0.05, **: *p* < 0.001.

The addition of exosomes to corneal stromal cell cultures produced statistically significant effects on cell proliferation. When exosomes produced by KC cells were added to healthy cells, no effects were observed at 24 h, but reductions in proliferation of about 10% and 14% were observed at 48 and 72 h, respectively. The addition of these same exosomes to KC cells also produced similar results, although in this case they could already be detected at 24 h. Curiously, the addition of exosomes isolated from healthy stromal cells produced a statistically significant reduction in the proliferation of around 10% in both healthy and keratoconic cells at 48 h (Figure 8A).

When the effect on corneal epithelial cells was analyzed, it was also observed that the addition of exosomes produced a reduction in cell proliferation, but this was dependent on the cellular origin of the exosomes. The exosomes produced by healthy cells did not produce a significant effect at either 24 or 48 h, but they did induce a reduction of around 16% in proliferation at 72 h. However, the exosomes isolated from the stromal cells of patients with KC produced a significant reduction in proliferation that increased as a function of time, reaching 14%, 30% and 33% at 24, 48 and 72 h, respectively. This reduction was also significant compared to that observed using exosomes isolated from healthy cells (Figure 8A).

When the effect on cell migration was studied, the results showed that the addition of exosomes produced by healthy stromal cells to both healthy stromal cells and those from patients with KC did not produce significant observable differences. In contrast, though, the addition of exosomes produced by KC cells increased migration significantly in both types of cells. The effect was particularly pronounced in healthy cells, around 80%, compared to KC cells, where migration increased by only 25% (Figure 8B). Furthermore, when the effect on cell migration of epithelial cells was observed, it was seen that that the addition of KC exosomes produced a significant increase compared to that observed in the control at 2 and 6 h, although the values did not reach statistical significance at 4 h. In contrast, healthy cell exosomes did not exhibit a significant increase in migration at 2 h, but did over longer periods of time, i.e., at both 4 and 6 h (Figure 8B).

## 4. Discussion

Exosomes are secreted by different types of cells and through specific molecular species, mainly proteins and different types of RNA, play key roles in tissue physiology and cell communication [30,31,32]. The molecular composition of exosomes is dependent on each cell type, but also on their physiological and pathological state, which makes these nanovesicles of great interest from the point of view of their potential application as diagnostic and therapeutic agents. In this sense, numerous studies have focused on exosomes in relation to cancer, cardiovascular diseases, autoimmune syndromes or neurodegenerative disorders [33]. However, the role of exosomes in relation to the normal physiology of the eye and eye diseases is poorly understood. While some studies have related them to the development of pathologies, such as glaucoma or age-related macular degeneration [32,50], there is a lack of data with respect to most pathologies, and specifically with KC, the most common type of degenerative eye disease.

Although the development of KC seems to affect all corneal layers to a greater or lesser extent, the greatest alterations are observed in the epithelium and stroma. The latter is particularly relevant because it constitutes 80% of the corneal thickness, and during the development of the pathology it becomes degraded, with keratocytes being observed to be at lower density and exhibiting an altered phenotype [4]. Given the role of exosomes in the structure and turnover of the ECM, as well as in cell communication, it is interesting to analyze them in stromal cells isolated from patients with KC and compare them to those of healthy individuals.

Exosomes isolated from corneal stromal cell cultures showed, both in healthy individuals and in KC patients, a mean particle size of 113 nm, which corresponds perfectly to the size of exosomes. However, in the case of healthy cells, a second group of vesicles with an apparent size of 178 nm was also identified. The appearance of bimodal distributions of vesicles when they are characterized by dynamic light scattering, with the presence of a second peak corresponding to sizes larger than expected, is, however, in line with previous descriptions, and corresponds to the potential presence of low levels of larger particles, which can alter the intensity-weighted size distribution due to their brighter light scattering, thus falsely magnifying the amount of large particles seen [51]. The characterization of the vesicles by an alternative procedure, namely TEM, allowed us to confirm and adjust the results, showing that the vast majority of the nanovesicles had sizes around 100 nm in both types of samples.

Proteomic analysis of exosomes isolated from healthy and KC stromal cell cultures allowed for the identification of 314 and 301 different proteins, respectively. These proteins included around two-thirds (31 out of 50) of those most commonly found in exosomes produced by other cell types, according to the ExoCarta database. Among the proteins identified in exosomes, frequently accepted markers of nanovesicles such as CD81 and various Hsp70 species were found, including HSPA2 in both types of exosomes, HSP1B in healthy ones, and HSPA5 and HSPA6 in keratoconus. CD9 was also detected, although it showed a differential expression, only being identified in exosomes of healthy cells at the level of detection used in this work. On the other hand, CD63 was not detected, which is in line with previous studies of the proteomic profile of the corneal tissues where this protein was also not detected [20], although in another recent study CD63 was detected in isolated extracellular vesicles of keratocytes, although its levels increased markedly when transformation into fibroblasts occurred [52], which supports the notion, as shown by our controls, that the cells maintain a stable phenotype.

However, it has recently been described that frequently biomarkers of exosomes are heterogeneous, and do not exhibit universal utility across different cell types. In this sense, certain tetraspanins have not been detected on exosomes produced by certain cell types, including CD9, CD81 or CD63, and it has been described that the low levels of certain tetraspanins at the cell surface of the exosomes mirrors a decreased expression of the cell. of origin [53].

Unbiased quantitative analysis has identified putative exosomal biomarkers and exclusion biomarkers. Among the former, the proteins have been grouped into classes on the bases of their measured abundance, with the first 4 classes being highly abundant proteins in exosomes, including SDCBP in class 1, SLC1A5, SLC3A2, GNB1 and CLTC in class 2, CD47, GNB2, ITGB1, BSG and B2M in 3, and ATP1A1, RAP1B and GNAI3 in 4. A total of 10 of these proteins could be detected in the exosomes produced by corneal stromal cells; SDCBP and CD47 were not detected, but these proteins could not be detected in previous studies of the complete proteome of corneal stromal cells, which would justify their absence [20], while RAP1B was the only protein with cellular expression not found in exosomes, which can be explained considering that the study of universal protein markers did not include cell types of certain tissues, including eye tissues [53]. On the other hand, 15 proteins consistently depleted in exosomes from different cellular sources that have been proposed as exosomal exclusion biomarkers have been described [53]. This group of proteins is preferably formed by nuclear proteins, and none of the 15 could be detected in the exosomes analyzed in this study, including HMGB1, HMGB2, HMGB3, NOLC1, SKP1, SERBP1, COX5B, SLIRP, PTMA, MAPRE1, PDAP1, EIF4B, EIF4H, PGM2, and the widely exclusion marker calnexin. A summary of these data is shown in Supplementary Table S7. Therefore, the analysis of molecular markers, both positive and negative, strongly supports the nature of isolated exosomes.

Previous studies have described the proteome corresponding to the three main layers of the cornea, including the stroma, in which 1679 different molecular species were identified [47]. The proteomic analysis of the exosomes allowed the identification of the presence of several of the most abundant proteins in the corneal stroma, although others were not detected. This data reinforces the idea that the molecular repertoire of proteins present in exosomes is specific, and dependent on the existence of mechanisms that control how they are sorted [28]. In this same sense, it is also significant that the content of exosomes includes only some of the adhesion and extracellular matrix proteins whose expression in the corneal stroma has been previously described, despite the role these nanovesicles play in relation to structure and turnover of the ECM, and the fact that this matrix comprises around 90% of stromal volume [48].

Among the proteins identified in the samples, the 79 whose expression was consistently detectable in all of the samples of the same type were selected and subjected to analysis, resulting in the detection of 10 whose levels presented statistically significant differences, of which 3 appeared over-expressed in the exosomes of KC cells (ACTB, SERPINE1 and HGFAC), and 7 in healthy cells (HRNR, CTNNB1, DSG1, GSN, LTF, GNAS and SERPINB3). Furthermore, 3 other proteins were detectable only in healthy samples (TRAPPC6A, AZGP1 and CSN2), and 1 in KC (PGK1). These proteins with altered levels were different from those previously described in proteomic studies of the corneal stroma in KC [1,19]. Moreover, and interestingly, although the transcriptional profile of corneal stromal cells derived from patients with KC has been previously described, detecting 423 differentially expressed genes, 187 down- and 236 up-regulated in KC-affected stroma [54], none of the proteins whose levels were found to be altered in this study corresponded to any of the genes whose transcriptional alteration had been reported in those previous studies on KC.

To give a functional meaning to the alterations observed in the proteins present in KC exosomes, the PANTHER classification system was used. Since proteins can fall into several different categories, the percentage values were normalized to a total value of 100%. Analysis was performed separately for proteins that were over- and under-expressed in KC. In the former, catalytic activities constituted the main functional group (50%), which dropped to 22.2% for under-expressed proteins, where hydrolase activities accounted for the majority (66.7%) compared to 40% in over-expressed proteins. Binding accounted for 33.3 and 44.4% of activity in the over- and under-expressed proteins, respectively, although in the former case they seem to be involved in heterogeneous activities, while in the latter they are related to binding to proteins or protein-containing complex (80%). Finally, one-sixth of the over-expressed proteins were involved in enzymatic regulation, while this increased to 33.3% in the under-expressed group, which, in addition, is mainly constituted by transcriptional regulators.

Although, as indicated above, the proteins with altered expression in exosomes found here do not correspond to the genes previously reported as having altered expression, it is possible to establish some functional similarities. Previous studies have linked genes with altered expression primarily to cell migration, collagen-containing ECM, adherens junction, as being intrinsic to the plasma membrane, cytokine and growth factor activity, growth factor binding, and kinase activity [54]. Several of the altered proteins found in this work are related to these activities, such as ACTB, SERPINE1, DSG and GSN (adherence and migration), as well as HGFAC, CTNNB1 and DSG1 (related to cytokine and growth factor activity), which would relate them to the progression of the pathology.

In order to better establish the involvement of the proteins that displayed altered expression in the KC, we carried out a functional association network study using the STRING program which included 89 genes that previous studies had related to the development of this pathology [8,55,56]. The altered molecules in the exosomes showed a variable relationship with the other species, although basically two groups appeared. One, formed by 4 proteins fundamentally involved in molecular binding and regulation, was directly associated with a dense central nucleus. The second, which presented various associations between its members, was located in a peripheral region, and seemed preferentially related to catalytic activities.

Along with proteins, an essential component of exosomes is the various types of non-coding RNAs, particularly miRNAs. Their importance is such that these molecules are thought to be the major contributor to the molecular events occurring in the recipient cell [57]. In this study it was possible to detect 800 different miRNAs, 692 present in exosomes isolated from healthy cells and 642 in pathological ones. A total of 370 of the miRNAs detected in this analysis of exosomes produced by healthy cells have been previously described in miRNA profile studies performed on whole human corneas. Although 131 of the species described in that work were not detected here, the current work did detect 322 miRNAs that were not found in this previous work [58]. Two of the most abundant species in this study, hsa-let-7a-5p and hsa-let-7f-5p, also appear among those most frequently identified the study on the whole cornea, while other species that were found in abundance here, did not figure as such in the previous work [58].

Analysis of the levels of the molecules consistently detected in all samples of the same type identified 16 miRNAs with statistically significant differences, 4 over-expressed in exosomes from healthy cells and 10 in those from KC, in addition to another 2 that were only detected in healthy samples. Previous studies have detected 12 miRNAs significantly downregulated in keratoconic corneal epithelia [59], although none of them correspond to those detected here in exosomes produced by stromal cells. Among the deregulated miRNAs is miR-184, which appears at levels 5 times higher in the exosomes produced by KC stromal cells compared to healthy ones. miR-184 is a molecule of interest, as it has been related to the expression of Pax6, a master regulator of eye development. Previous studies have shown that the knockdown of miR-184 results in a decrease in Pax6, and its deregulation has been associated with corneal diseases [60,61]. In addition, it has been described that a mutation altering the seed region of miR-184 is responsible for familial severe KC combined with cataract [49].

Bioinformatic analyses of genes potentially regulated by deregulated miRNAs identified 1121 genes potentially regulated by 1 miRNA species with differential expression, 126 by 2 of them, 8 by 3, and 1 by 4. These genes were involved in numerous biological functions, mainly binding, catalytic activity and molecular function regulation, while at least one of them, miR-2355-3p, is potentially involved in the post-transcriptional regulation of the gene encoding one of the proteins with altered expression in KC exosomes, SERPINE1. Another 7 miRNAs also appeared to be related to the regulation of genes previously identified in relation to KC.

The molecules encapsulated by exosomes are capable of modifying the cellular behavior of target cells, which indicates they have pleiotropic functions, including differentiation, inflammation, immunosuppression, neurogenesis and angiogenesis. Some studies have analyzed the potential applications of exosome-based therapies in ocular diseases, including ocular neo-vascularization diseases, macular degeneration and glaucoma [32,62,63]. Given the differences observed in the composition of proteins and miRNAs between exosomes produced by healthy corneal stromal cells and those of KC patients, we approached the study of the influence that the addition of these nanovesicles could have on cell behavior in order to explore their potential from the perspective of future therapeutic developments.

Among the genes predicted as biological targets of deregulated miRNAs, certain encoding PGs were also identified. These molecules play important roles in relation to biogenesis, secretion and composition of exosomes, as well as in their internalization by the receiving cell and their functional activity within it [27]. PGs act as specific catalysts for interaction with a multitude of ligands, resulting in them having a wide range of essential effects on cell physiology [23]. Furthermore, these functions are highly dependent on the structure of the glycosaminoglycan chains present in these molecules, which in turn is finely regulated by numerous genes whose expression is altered in pathological processes, including KC [21]. The sensitivity that the expression of these genes shows with respect to physiological and pathological conditions makes them a good model to observe the effect of the addition of exosomes on cell transcription. The results showed a variable effect that depended on the concentration of exosomes added. The deviation in the transcription values observed in KC with respect to healthy cells increased with the addition of the nanovesicles at a concentration of 120 µg protein/mL, then experienced a relative normalization at 240 µg/mL, and a new deviation when exosomes were added at 480 µg/mL. The dose-dependent effect of the addition of extracellular vesicles has been previously observed in other studies of the eye [64], and these results point to the possibility that an appropriate concentration of exosomes is capable of restoring, at least partially, normal transcription levels in pathological cells.

The influence of the addition of exosomes on the migration and proliferation of corneal stromal cells was also analyzed. The addition of exosomes produced by KC cells induced a reduction in cell proliferation in both healthy and KC cells, although it was more pronounced in the latter. Reductions in exosome-induced proliferation of healthy cells were also observed but was limited to analyses after 48 h of incubation. The effect was much more notable on migration, though, where KC exosomes produced a marked increase, particularly after being added to healthy cells. However, healthy exosomes did not produce any detectable effect. These results show a clear differential influence of pathological exosomes on cell behavior compared to that produced by healthy cells. The existence of notable differences in the effect produced by the addition of exosomes from healthy cells with respect to pathological ones has also been described in other situations, for example those produced by normal and diabetic corneolimbal keratocytes [65].

Bowman’s membrane seems to constitute a barrier for the transit of exosomes towards the epithelium, distinct from Descemet’s membrane [66]. However, Bowman’s membrane is altered in KC, including the presence of ruptures and infiltrated cellular elements [2,4]. These alterations would potentially allow the exosomes produced by the stromal cells to reach the epithelium, which is why we also analyzed the alterations induced on the behavior of the corneal epithelial cells. Both exosomes produced by healthy stromal cells and those from KC induced a reduction in epithelial cell proliferation, although this was notably greater in the case of the KC stromal cells. Migration, for its part, experienced an increase, which was detected earlier than the effect on proliferation, after the addition of pathological exosomes. Together, these data show an alteration in the cellular behavior of the epithelium promoted by stromal exosomes which could be related to some of the alterations observed in it in relation to the development of the pathology [2,4].

## 5. Conclusions

In conclusion, the exosomes produced by corneal stromal cells from patients with KC demonstrate molecular differences with respect to those produced by healthy cells, which give them a differential capacity to alter the gene expression and behavior of the cells on which they act. These results also point to possibilities for future therapeutic developments.

## Figures and Tables

**Table 1:** Proteins detectable in all exosome samples isolated from either healthy stromal cells or those from patients with KC.

Protein Symbol	UniPROT	Description/Name	Keratoconus ^1^	Normal ^1^
A2M	H0YFH1	Alpha-2-macroglobulin	0.143755	0.117508
ACTB	G5E9R0	Actin, cytoplasmic 1	3.044770	0.954318
ACTBL2	Q562R1	Beta-actin-like protein 2	0.472427	0.416883
AFP	J3KMX3	Alpha-fetoprotein	0.301108	0.197081
AHSG	C9JV77	Alpha-2-HS-glycoprotein	0.652708	0.543988
ANXA1	Q5T3N0	Annexin A1	0.952801	0.793662
ANXA2	H0YNB8	Annexin A2	4.321061	4.004153
ANXA5	D6RBE9	Annexin A5	1.281670	1.428309
APOA1	F8W696	Apolipoprotein A-I	0.243717	0.184766
ART4	H7C2G2	NAD(P)(+)--arginine ADP-ribosyltransferase	0.211927	0.191949
AZGP1	H7BZJ8	Zinc-alpha-2-glycoprotein	0.144309	0.000000
C3	M0QYC8	Complement C3	0.271774	0.252169
C4A	A0A140TA32	Complement C4-A	0.104644	0.090665
CD44	H0YE40	CD44 antigen	0.390023	0.203275
CLEC3B	E9PHK0	Tetranectin	0.732630	0.423420
COL1A2	A0A087WTA8	Collagen alpha-2(I) chain	0.232500	0.127181
COL5A1	H7BY82	Collagen alpha-1(V) chain	0.028984	0.076454
COL6A1	P12109	Collagen alpha-1(VI) chain	0.444436	0.251104
COMP	G3XAP6	Cartilage oligomeric matrix protein	0.088188	0.056545
CTNNB1	C9IZ65	Catenin beta-1	0.048813	0.114816
CTSD	A0A7I2V2N3	Cathepsin D	0.349379	0.472076
DCD	P81605	Dermcidin	0.819703	0.595040
DSC1	Q08554	Desmocollin-1	0.086888	0.065345
DSG1	Q02413	Desmoglein-1	0.183173	0.328223
DSP	P15924	Desmoplakin	0.116647	0.199771
ENO1	K7ERS8	Alpha-enolase	0.684927	0.537460
FBLN1	B1AHM9	Fibulin-1	0.114234	0.150116
FLG2	Q5D862	Filaggrin-2	0.022611	0.028105
FN1	H0Y7Z1	Fibronectin	0.553519	0.321627
GAPDH	P04406	Glyceraldehyde-3-phosphate dehydrogenase	1.253172	1.073777
GC	D6RF35	Vitamin D-binding protein	0.421269	0.310700
GNA13	Q14344	Guanine nucleotide-binding protein subunit alpha-13	0.148349	0.108959
GNAS	A0A590UJ47	Guanine nucleotide-binding protein G(s) subunit alpha isoforms XLas	0.583043	1.155494
GSN	Q5T0H8	Gelsolin	0.208645	0.369956
HBA1	P69905	Hemoglobin subunit alpha	1.045289	1.310054
HGFAC	D6RAR4	Hepatocyte growth factor activator	0.103574	0.026398
HRNR	Q86YZ3	Hornerin	0.013325	0.040701
HSP90AA1	G3V2J8	Heat shock protein HSP 90-alpha	0.091852	0.117342
ITIH2	Q5T987	Inter-alpha-trypsin inhibitor heavy chain H2	0.190179	0.122103
ITIH3	E7ET33	Inter-alpha-trypsin inhibitor heavy chain H3	0.112654	0.136175
JUP	C9JPI2	Junction plakoglobin	0.342050	0.727787
LTF	E7EQB2	Lactotransferrin	0.227806	0.382644
LUM	P51884	Lumican	0.169542	0.164988
MFGE8	H0YKS8	Lactadherin	0.516690	0.682248
MSN	P26038	Moesin	0.314202	0.178213
ODF2	S4R462	Outer dense fiber protein 2	0.029924	0.028792
PRDX2	A6NIW5	Peroxiredoxin-2	0.706698	0.840749
PRDX4	A6NG45	Peroxiredoxin-4	0.614590	0.491306
PTX3	P26022	Pentraxin-related protein PTX3	0.530573	0.309626
PZP	P20742	Pregnancy zone protein	0.059333	0.059923
RBP4	Q5VY30	Retinol binding protein 4, plasma, isoform CRA_b	0.242353	0.167108
RPL4	P36578	60S ribosomal protein L4	0.054390	0.076779
S100A7	P31151	Protein S100-A7	1.077940	1.636534
S100A8	P05109	Protein S100-A8	1.135480	1.176553
S100A9	P06702	Protein S100-A9	0.497486	1.321336
SERPINA7	P05543	Thyroxine-binding globulin	0.210750	0.244745
SERPINB12	Q96P63	Serpin B12	0.177998	0.291514
SERPINB3	P29508	Serpin B3	0.353509	1.231848
SERPINC1	P01008	Antithrombin-III	0.581404	0.580876
SERPINE1	P05121	Plasminogen activator inhibitor 1	0.572236	0.286820
SERPINF1	A0A0J9YXF9	Pigment epithelium-derived factor	0.270115	0.268728
SERPINF2	A0A0J9YY65	Alpha-2-antiplasmin	0.137753	0.103781
SLC1A5	M0R144	Neutral amino acid transporter B(0)	0.266010	0.215608
SLC3A2	A0A7P0Z4P5	4F2 cell-surface antigen heavy chain	0.235325	0.251178
TF	P02787	Serotransferrin	0.288676	0.193706
TGM3	Q08188	Protein-glutamine gamma-glutamyltransferase E	0.303941	0.287802
THBS1	A8MZG1	Thrombospondin-1	0.258995	0.222754
THY1	J3QRJ3	Thy-1 membrane glycoprotein	0.441508	0.244056
TIMP1	Q5H9B5	Metalloproteinase inhibitor 1	1.168873	0.669724
TRAPPC6A	O75865	Trafficking protein particle complex subunit 6A	0.000000	0.163156
TUBA1C	Q9BQE3	Tubulin alpha-1C chain	0.470637	0.439214
TUBB	P07437	Tubulin beta chain	0.215058	0.256739
TXN	P10599	Thioredoxin	0.306628	0.249533
VASH1	Q7L8A9	Vasohibin-1	0.074175	0.067182
VIM	B0YJC4	Vimentin	0.262035	0.516628
YWHAE	I3L3T1	14-3-3 protein epsilon	0.250273	0.310520

^1^ Normalized emPAI.

**Table 2:** Genes previously related to KC that are potentially regulated by miRNAs whose levels appear altered in KC stromal cell exosomes.

miRNA	Protein Symbol	UniPROT	Description/Name
hsa-miR-2355-3p	ACTN1	P12814	Alpha-actinin-1
hsa-miR-183-5p	FNDC3B	Q53EP0	Fibronectin type III domain-containing protein 3B
hsa-miR-455-3p	NFIB	O00712	Nuclear factor 1 B-type
hsa-miR-3377	SORL1	Q92673	Sortilin-related receptor
hsa-miR-3192-5p	TIMP3	P35625	Metalloproteinase inhibitor 3
hsa-miR-34a-3p	TNF	P01375	Tumor necrosis factor
hsa-miR-183-5p	ZEB1	P37275	Zinc finger E-box-binding homeobox 1

## Data Availability

The data presented in this study are contained within the article or supplementary material.

## Supplementary Materials

The following supporting information can be downloaded at: https://www.mdpi.com/2227-9059/10/10/2348/s1, Figure S1: Alteration in gene expression of genes encoding HSPG and SLRP in KC cells induced by exosomes produced by healthy stromal cells; Table S1: List of proteins identified by LC-MS/MS in exosomes isolated from healthy stromal cell cultures and in isolates from keratoconus stromal cell cultures; Table S2: Determination of the presence of the 50 main proteins most frequently identified in exosomes (http://exocarta.org/exosome_markers_new; accessed on 17 June 2021) in exosomes isolated from healthy corneal stroma and keratoconus cells; Table S3: List of miRNAs identified by Next-Generation Sequencing in exosomes isolated from healthy stromal cell cultures and in isolates from keratoconus stromal cell cultures; Table S4: List of miRNAs species that appeared consistently in all samples of the same type; Table S5: Prediction of the biological targets of miRNAs whose expression appears altered in exosomes of corneal cells from patients with keratoconus; Table S6: Genes that encode PG core proteins, or are related to the GAG chains covalently linked to them, predicted as biological targets of miRNAs whose levels appear altered in the exosomes of keratoconic cells
