# Supplementary material for: Exosomes Released by Corneal Stromal Cells Show Molecular Alterations in Keratoconus Patients and Induce Different Cellular Behavior
Source: Biomedicines. 2022 Sep 21;10(10):2348. doi: 10.3390/biomedicines10102348 (PMC9598276; doi:10.3390/biomedicines10102348)

Supplementary Figure S1.

Human corneal stromal cells in culture. Image A corresponds to a passage 3 culture of healthy human corneal stromal cells (non keratoconus control). Image B corresponds to a passage 3 culture of human keratoconus corneal stromal cells. Image C corresponds to a passage 6 culture of human keratoconus corneal stromal cells. Note the absence of αSMA-positive cells in A and B and the presence of positive elements (in green) in the passage 6 culture (C). (Scale bars 20 µm).

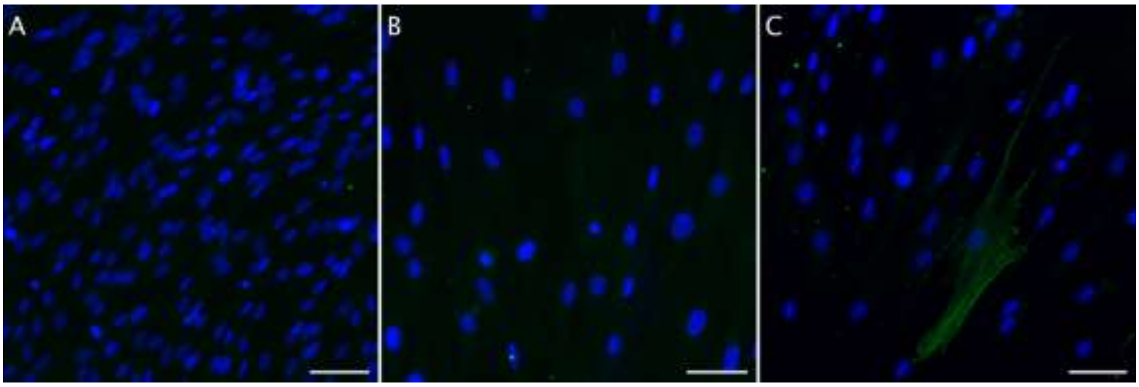

Supplement: Supplementary file 1 [file biomedicines-10-02348-s001.zip › biomedicines-1778042-supplementary/Supplementary Figure S1.pdf]
