# Supplementary material for: Exosomes Released by Corneal Stromal Cells Show Molecular Alterations in Keratoconus Patients and Induce Different Cellular Behavior
Source: Biomedicines. 2022 Sep 21;10(10):2348. doi: 10.3390/biomedicines10102348 (PMC9598276; doi:10.3390/biomedicines10102348)

Supplementary Figure S2.

Alterations in the transcription of genes encoding heparan sulfate proteoglycans (HSPGs) and small leucine-rich proteoglycans (SLRPs). Relative Abundances for healthy corneal stromal cells (blue bars) and for corneal stromal cells from KC patients cultured alone (orange bars) or in the presence of exosomes isolated from healthy cell cultures at concentrations of 120, 240, and 480 µg protein/ml are plotted (green, purple and grey bars respectively). Values on the Y axis are plotted on a logarithmic scale, and spreads represent the standard deviation.

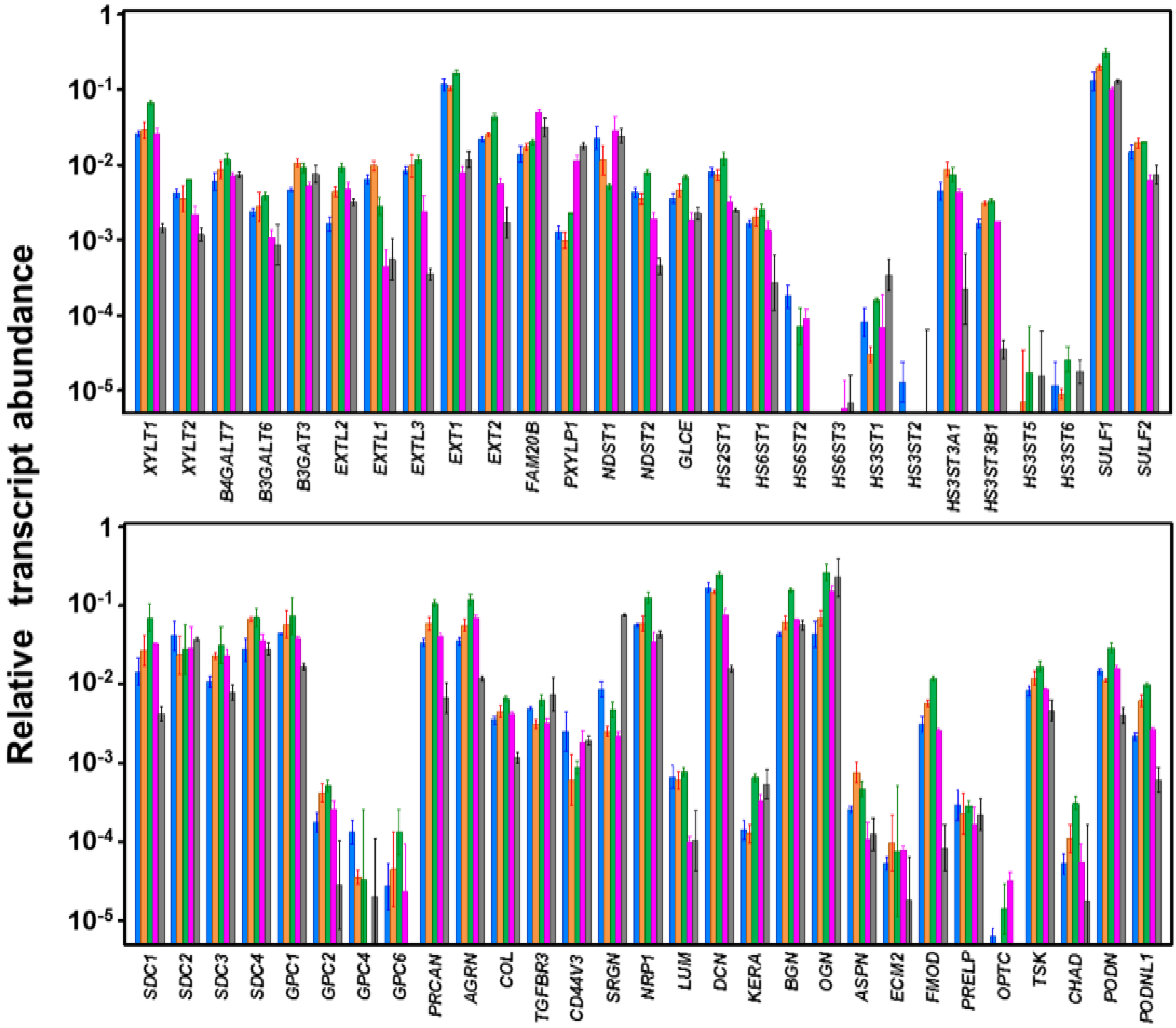

Supplement: Supplementary file 1 [file biomedicines-10-02348-s001.zip › biomedicines-1778042-supplementary/Supplementary Figure S2.pdf]
