# Supplementary material for: Exosomes Released by Corneal Stromal Cells Show Molecular Alterations in Keratoconus Patients and Induce Different Cellular Behavior
Source: Biomedicines. 2022 Sep 21;10(10):2348. doi: 10.3390/biomedicines10102348 (PMC9598276; doi:10.3390/biomedicines10102348)
Supplement: Supplementary file 1 [file biomedicines-10-02348-s001.zip › biomedicines-1778042-supplementary/Supplementary Table S1.pdf]

Supplementary Table S1

List of proteins identified by LC-MS/MS in exosomes isolated from healthy stromal cell cultures and in isolates from keratoconus stromal cell cultures.

Protein Symbol	UniPROT	Healthy Stroma	KC Stroma
**A1BG**	M0R009	✓	✓
**A2M**	H0YFH1	✓	✓
**A2ML1**	F5GXP1	✓	✓
**ACTA2**	F6QUT6	✓	
**ACTB**	G5E9R0	✓	✓
**ACTBL2**	Q562R1	✓	✓
**ACTC1**	P68032	✓	✓
**ACTG1**	A0A7P0TBL1	✓	✓
**ACTN1**	G3V2W4	✓	✓
**ACTN4**	K7EP19	✓	
**ACTR3**	B4DXW1		✓
**ADGB**	H0YC38		✓
**ADH5**	D6RAY0	✓	
**AFM**	P43652	✓	✓
**AFP**	J3KMX3	✓	✓
**AHCY**	P23526	✓	✓
**AHSG**	C9JV77	✓	✓
**ALDOA**	J3KPS3	✓	✓
**ALOX12B**	A0A3B3IRK2		✓
**AMY1A**	Q5T084		✓
**ANKRD20A4**	Q4UJ75		✓
**ANPEP**	H0YMC1	✓	✓
**ANXA1**	Q5T3N0	✓	✓
**ANXA11**	H0Y6E1	✓	
**ANXA2**	H0YNB8	✓	✓
**ANXA4**	B4E1S2	✓	
**ANXA5**	D6RBE9	✓	✓
**ANXA6**	E5RIU8	✓	✓
**APOA1**	F8W696	✓	✓
**APOB**	A0A669KB70		✓
**APOD**	C9JX71		✓
**APOH**	J3KS17	✓	✓
**ARF3**	F5H6T5	✓	
**ARG1**	A0A5F9ZGY6	✓	✓
**ARHGDIA**	J3KTF8	✓	
**ARHGEF37**	D6RJH4	✓	
**ART4**	H7C2G2	✓	✓
**ASAH1**	A0A1B0GVA3	✓	✓
**ATP1A1**	Q5TC02	✓	✓
**ATP1B3**	H7C4L9	✓	
**ATP2B4**	H7BZS8	✓	
**ATP5A1**	P25705	✓	✓
**ATP5B**	Q0QEN7	✓	✓
**ATP6V1H**	E5RG49	✓	✓
**ATR**	A0A590UJ01		✓
**ATRX**	A0A096LNX6		✓
**AZGP1**	H7BZJ8	✓	✓
**B2M**	F5H6I0		✓
**B4GAT1**	O43505		✓
**BASP1**	U3KQP0	✓	
**BLMH**	K7EMJ3	✓	✓
**BSG**	A0A087WUV8	✓	
**C1R**	B4DPQ0	✓	
**C1S**	A0A087X232	✓	✓
**C3**	M0QYC8	✓	✓
**C4A**	A0A140TA32	✓	✓
**C5**	P01031		✓
**C7**	P10643	✓	✓
**C9**	P02748	✓	✓

**CALML5**	Q9NZT1	✓	
**CAND1**	A0A0C4DGH5		✓
**CAPN1**	E9PLX0	✓	
**CAPZA2**	F8WED3	✓	
**CASP14**	P31944		✓
**CAT**	A0A3B3ITJ0		✓
**CAV1**	C9JKI3	✓	
**CD151**	E9PLZ6	✓	
**CD44**	H0YE40	✓	✓
**CD81**	E9PM31	✓	✓
**CD9**	A0A7I2V3T0	✓	
**CDSN**	Q2L6G8	✓	✓
**CES4A**	A0A0C4DGH1		✓
**CFAP100**	E9PQV5	✓	
**CFI**	G3XAM2	✓	✓
**CFL1**	G3V1A4	✓	✓
**CFL2**	F8WDN3	✓	
**CHIA**	Q5VUV5	✓	✓
**CHN2**	H7C138		✓
**CKM**	P06732		✓
**CLCF1**	Q9UBD9	✓	
**CLEC3B**	E9PHK0	✓	✓
**CLIC1**	O00299	✓	✓
**CLIC4**	Q9Y696	✓	
**CLTC**	J3KSQ2	✓	
**CLU**	H0YLK8	✓	✓
**CNOT3**	H0Y5X7	✓	
**COL11A1**	A0A2R8Y5N4	✓	✓
**COL1A1**	I3L3H7	✓	✓
**COL1A2**	A0A087WTA8	✓	✓
**COL3A1**	H7C435	✓	✓
**COL5A1**	H7BY82	✓	✓
**COL6A1**	P12109	✓	✓

**COL6A2**	H7C0M5		✓
**COL6A3**	I3L392	✓	
**COLEC12**	Q5KU26	✓	
**COMP**	G3XAP6	✓	✓
**COX5B**	P10606		✓
**CPA4**	C9J7D6	✓	✓
**CREBBP**	A0A1B0GUU0	✓	
**CREG1**	A0A3B3IRL2	✓	
**CSN1S1**	D6RF34	✓	✓
**CSN3**	P07498		✓
**CSTA**	C9J0E4		✓
**CTNNB1**	C9IZ65	✓	✓
**CTSA**	A0A7I2V504	✓	
**CTSD**	A0A7I2V2N3	✓	✓
**DCD**	P81605	✓	✓
**DCN**	F8VSI3	✓	
**DDX52**	A0A087X1P7	✓	
**DEFA1**	P59665		✓
**DES**	P17661	✓	
**DHCR7**	E9PLZ2		✓
**DSC1**	Q08554	✓	✓
**DSC3**	J3QRL9	✓	✓
**DSG1**	Q02413	✓	✓
**DSP**	P15924	✓	✓
**ECM1**	Q16610	✓	✓
**EEF1A1**	A0A7I2V3H3	✓	✓
**EEF1A1P5**	Q5VTE0	✓	✓
**EEF2**	M0R0I6	✓	✓
**EFEMP1**	Q580Q6	✓	✓
**EHD1**	C9J2Z4	✓	
**EIF4A2**	J3KSN7	✓	✓
**ENO1**	K7ERS8	✓	✓
**ENO2**	U3KQQ1	✓	✓

**ENO3**	E5RI09	✓	✓
**EPM2A**	A0A1W2PRC9		✓
**ERVV-2**	B6SEH9		✓
**EZR**	E7EQR4		✓
**F10**	F8WBM7	✓	✓
**F2**	E9PIT3	✓	✓
**FABP5**	I6L8B7		✓
**FBLN1**	B1AHM9	✓	✓
**FBXO21**	H0YIE9		✓
**FGB**	D6REL8		✓
**FGG**	C9JPQ9		✓
**FLG2**	Q5D862	✓	✓
**FLNA**	A0A7I2V3E6	✓	
**FLNB**	H7C5L4	✓	
**FN1**	H0Y7Z1	✓	✓
**FPGS**	Q5JU23	✓	
**FSTL1**	H7C4W4	✓	✓
**G6PD**	P11413	✓	✓
**GALNS**	Q6MZF5	✓	
**GAPDH**	P04406	✓	✓
**GAPDHS**	K7EMB2	✓	
**GBA**	A0A0G2JLB3	✓	
**GC**	D6RF35	✓	✓
**GDI2**	V9GYJ7	✓	
**GFAP**	B4DIR1	✓	✓
**GFM2**	Q969S9	✓	
**GGCT**	H7BZK5		✓
**GGH**	A0A7I2V5P2		✓
**GNA12**	C9J2Y7		✓
**GNA13**	Q14344	✓	✓
**GNAI2**	F8WBG4	✓	✓
**GNAI3**	P08754	✓	✓
**GNAO1**	A0A1W2PRE1		✓

**GNAS**	A0A590UJ47	✓	✓
**GNAT1**	C9JCV8		✓
**GNAT2**	A0A087WZE5		✓
**GNB1**	B3KVK2	✓	
**GNB2**	E7EP32	✓	✓
**GNB4**	A0A6Q8PFV8	✓	✓
**GNG12**	Q9UBI6	✓	✓
**GPI**	K7EIL4	✓	
**GSDMA**	J3KRG2	✓	✓
**GSN**	Q5T0H8	✓	✓
**GSTP1**	A8MX94	✓	
**HAL**	H0YHB0	✓	✓
**HBA1**	P69905	✓	✓
**HBA2**	G3V1N2		✓
**HBD**	E9PEW8	✓	✓
**HBE1**	P02100	✓	✓
**HBG2**	P69892		✓
**HERC1**	H0YK60		✓
**HGFAC**	D6RAR4	✓	✓
**HIST1H2BN**	Q99877		✓
**HIST2H3A**	Q71DI3		✓
**HLA-A**	A0A0G2JL56	✓	
**HMCN1**	Q5TCP6		✓
**Homo sapiens**		✓	✓
**Homo sapiens**		✓	✓
**Homo sapiens**		✓	✓
**Homo sapiens**			✓
**HPX**	Q9BS19	✓	✓
**HRNR**	Q86YZ3	✓	✓
**HSP90AA1**	G3V2J8	✓	✓
**HSP90AB1**	P08238		✓
**HSP90AB2P**	Q58FF8		✓
**HSP90B1**	A0A7P0T823	✓	✓

**HSPA1B**	A0A0G2JIW1	✓	
**HSPA1L**	Q53FA3	✓	✓
**HSPA2**	P54652	✓	✓
**HSPA5**	A0A7P0TB36		✓
**HSPA6**	P17066		✓
**HSPA8**	E9PKE3	✓	✓
**HSPB1**	A0A6Q8PGY2		✓
**HSPG2**	H0Y5A9		✓
**HUNK**	P57058	✓	✓
**IDH1**	C9J4N6		✓
**IGFBP6**	F8VVA5	✓	✓
**IGHG1**	A0A0A0MS08	✓	✓
**IGHG2**	A0A286YEY4	✓	✓
**IGHG3**	A0A4W9A917	✓	
**IGKC**	P01834	✓	✓
**IGKV3D-20**	A0A0C4DH25	✓	
**IGLC2**	P0DOY2	✓	✓
**IGLL5**	A0A0B4J231	✓	✓
**IMPA2**	K7ELF8	✓	
**IQGAP1**	H0YKA5	✓	
**IRAK1**	H7C1F0		✓
**ITGA2**	D6RG08	✓	✓
**ITGAV**	P06756	✓	
**ITGB1**	E7EQW5	✓	✓
**ITIH1**	H7C5I0	✓	✓
**ITIH2**	Q5T987	✓	✓
**ITIH3**	E7ET33	✓	✓
**ITIH4**	H7C0L5	✓	✓
**IYD**	F6VN83		✓
**JUP**	C9JPI2	✓	✓
**KCTD12**	Q96CX2	✓	
**KLK7**	P49862	✓	
**KPRP**	Q5T749	✓	✓

**KRT222**	J3QQR9	✓	
**KRT23**	Q9UFN7	✓	✓
**KRT35**	C4AM86		✓
**LALBA**	F8VWU1		✓
**LAMP1**	P11279	✓	✓
**LCN1**	P31025		✓
**LCN1P1**	Q5VSP4	✓	✓
**LDHA**	F5GXU1	✓	✓
**LDHB**	A0A5F9ZHM4	✓	✓
**LGALS1**	F8WEI7	✓	✓
**LGALS7**	M0R281		✓
**LOC100653049**		✓	✓
**LPAR1**	B1AP63	✓	
**LSM4**	M0QXB0		✓
**LTF**	E7EQB2	✓	✓
**LUM**	P51884	✓	✓
**LYZ**	P61626	✓	✓
**MAGT1**	Q9H0U3	✓	
**MARCKS**	P29966	✓	✓
**MDH1**	C9JF79	✓	
**MDH2**	G3XAL0	✓	
**MFGE8**	H0YKS8	✓	✓
**MMP2**	H3BV48	✓	✓
**MSH3**	A0A590UJW0	✓	
**MSN**	P26038	✓	✓
**MTDH**	H0YBE0		✓
**MVP**	H3BUP3	✓	
**MYH14**	A1L2Z2	✓	
**MYH9**	Q5BKV1	✓	
**NCCRP1**	Q6ZVX7		✓
**NCSTN**	Q5T210	✓	
**NEFH**	P12036	✓	✓
**NEK5**	A0A3B3ITQ6		✓

**NPFFR1**	Q9GZQ6	✓	✓
**NQO1**	P15559		✓
**NT5E**	H0Y3X5	✓	✓
**ODF2**	S4R462	✓	✓
**OGDH**	Q02218		✓
**OGFOD3**	K7ELR2		✓
**P2RX3**	A0A087X210		✓
**PADI1**	Q9ULC6	✓	
**PCLO**	E9PE96		✓
**PCOLCE**	Q15113	✓	
**PCYOX1**	F8W8W4	✓	
**PEPD**	V9GYL0	✓	
**PFN1**	P07737	✓	✓
**PGAM1**	P18669	✓	
**PGAM4**	Q8N0Y7	✓	✓
**PGD**	K7ELN9	✓	
**PGK1**	P00558		✓
**PIGR**	P01833	✓	
**PIP**	P12273	✓	✓
**PKM**	B4DNK4	✓	✓
**PLA2G4B**	H3BSX5	✓	
**PLBD1**	F5H053	✓	
**PLEC**	Q15149		✓
**PLG**	P00747	✓	✓
**PLPP3**	O14495	✓	
**PNP**	G3V2H3	✓	
**POGK**	Q9P215	✓	
**POTEE**	Q6S8J3		✓
**POTEF**	A5A3E0	✓	
**PPARD**	Q03181	✓	
**PPIA**	A0A7I2V5J5	✓	✓
**PRDX1**	A0A0A0MSI0	✓	✓
**PRDX2**	A6NIW5	✓	✓
**PRDX4**	A6NG45	✓	✓
**PRDX6**	P30041		✓
**PRSS1**	E7EQ64		✓
**PSMA2**	A0A024RA52	✓	
**PSMB3**	A0A087WUL2	✓	
**PSMB4**	P28070	✓	
**PSMB5**	H0YJM8	✓	
**PSMB6**	I3L3X7	✓	✓
**PTX3**	P26022	✓	✓
**PZP**	P20742	✓	✓
**QSOX1**	O00391	✓	
**RAB10**	P61026		✓
**RAB15**	G3V562	✓	✓
**RAB1C**	Q92928	✓	✓
**RAB33B**	Q9H082	✓	✓
**RAB35**	Q15286	✓	✓
**RAB37**	Q96AX2	✓	
**RAB3A**	M0R257	✓	✓
**RAB6B**	Q9NRW1	✓	✓
**RAB7A**	C9J4S4	✓	
**RAC2**	B1AH79	✓	
**RBP4**	Q5VY30	✓	✓
**RDX**	A0A2R8Y4H6	✓	✓
**RNH1**	E9PIK5	✓	
**ROCK2**	O75116	✓	
**ROS1**	H0Y3T9		✓
**RPL4**	P36578	✓	✓
**RPL9**	H0Y9V9		✓
**RPS2**	E9PPT0		✓
**RPS3**	H0YF32	✓	✓
**RPS5**	M0R0R2		✓
**RRAS**	P10301	✓	
**RTN4**	F8W914	✓	✓

**S100A11**	P31949		✓
**S100A14**	Q9HCY8		✓
**S100A6**	R4GN98	✓	✓
**S100A7**	P31151	✓	✓
**S100A8**	P05109	✓	✓
**S100A9**	P06702	✓	✓
**SASS6**	Q6UVJ0		✓
**SBSN**	Q6UWP8		✓
**SDR9C7**	Q8NEX9	✓	
**SERPINA12**	Q8IW75	✓	
**SERPINA7**	P05543	✓	✓
**SERPINB1**	P30740	✓	
**SERPINB12**	Q96P63	✓	✓
**SERPINB3**	P29508	✓	✓
**SERPINB4**	C9JZ65	✓	✓
**SERPINB7**	C9JM00	✓	
**SERPINC1**	P01008	✓	✓
**SERPIND1**	P05546	✓	
**SERPINE1**	P05121	✓	✓
**SERPINF1**	A0A0J9YXF9	✓	✓
**SERPINF2**	A0A0J9YY65	✓	✓
**SFN**	P31947	✓	✓
**SH3RF1**	D6RAL3		✓
**SHROOM3**	A0A2R8Y5P9	✓	✓
**SLC16A3**	J3QQS9	✓	✓
**SLC1A5**	M0R144	✓	✓
**SLC25A31**	Q9H0C2	✓	✓
**SLC25A5**	P05141	✓	✓
**SLC25A6**	P12236		✓
**SLC2A1**	A0A6Q8PFI8		✓
**SLC3A2**	A0A7P0Z4P5	✓	✓
**SOX10**	A6PVD3		✓
**SPARC**	E5RJA5	✓	✓
**SPP2**	Q13103	✓	
**STOM**	F8VSL7	✓	✓
**STOML3**	Q8TAV4	✓	
**SYPL1**	Q16563	✓	✓
**TAF1A**	Q15573	✓	
**TAGLN**	E9PJ32	✓	
**TALDO1**	E9PM01	✓	
**TF**	P02787	✓	✓
**TGFBI**	H0Y8L3	✓	✓
**TGM1**	H0YNM4	✓	✓
**TGM3**	Q08188	✓	✓
**TGM5**	O43548	✓	
**THBS1**	A8MZG1	✓	✓
**THY1**	J3QRJ3	✓	✓
**TIMP1**	Q5H9B5	✓	✓
**TKT**	F8WAX4		✓
**TMCC1**	Q6N039	✓	✓
**TNFAIP8L1**	Q8WVP5		✓
**TPI1**	U3KPS5		✓
**TRAJ56**	A0A075B6Z2	✓	
**TRAP1**	I3L4L7	✓	✓
**TRAPPC6A**	O75865	✓	
**TRBV4-1**	A0A0J9YWV2	✓	
**TSNARE1**	A0A590UJA6		✓
**TTBK2**	H3BQ25	✓	
**TUBA1C**	Q9BQE3	✓	✓
**TUBA3E**	Q6PEY2		✓
**TUBA4A**	P68366	✓	
**TUBA4B**	Q9H853	✓	
**TUBA8**	Q9NY65	✓	
**TUBB**	P07437	✓	✓
**TUBB8**	Q5SQY0		✓
**TXK**	P42681		✓

**TXN**	P10599	✓	✓
**TXNRD1**	A0A087WSY9	✓	
**TYMP**	C9JGI3	✓	
**UBA52**	M0R1M6	✓	✓
**UBAC2**	A0A087WXT1	✓	✓
**UBB**	B4DV12		✓
**UCHL1**	P09936	✓	✓
**VASH1**	Q7L8A9	✓	✓
**VAT1**	K7EJM4		✓
**VIM**	B0YJC4	✓	✓
**VNN1**	O95497	✓	✓
**VNN2**	E9PRD9		✓
**VTN**	P04004		✓
**WDR1**	O75083	✓	
**WDR60**	Q8WVS4		✓
**YWHAB**	Q4VY20		✓
**YWHAE**	I3L3T1	✓	✓
**YWHAG**	P61981	✓	
**YWHAH**	F8WEB6	✓	
**YWHAQ**	E9PG15		✓
**YWHAZ**	B7Z2E6	✓	✓
**ZG16B**	I3L1H9	✓	✓
**ZNF268**	F5H670	✓	
**ZNF462**	H3BLX4		✓
**ZNF609**	E7ERY8		✓
	TOTAL:	**314**	**301**
