# Supplementary material for: Exosomes Released by Corneal Stromal Cells Show Molecular Alterations in Keratoconus Patients and Induce Different Cellular Behavior
Source: Biomedicines. 2022 Sep 21;10(10):2348. doi: 10.3390/biomedicines10102348 (PMC9598276; doi:10.3390/biomedicines10102348)
Supplement: Supplementary file 1 [file biomedicines-10-02348-s001.zip › biomedicines-1778042-supplementary/Supplementary Table S2.pdf]

Supplementary Table S2

Determination of the presence of the 50 main proteins most frequently identified in exosomes (http://exocarta.org/exosome_markers_new) in exosomes isolated from healthy corneal stroma and keratoconus cells

		**Normal**	**Keratoconus**
1	CD9	✓	
2	HSPA8	✓	✓
3	PDCD6IP		
4	GAPDH	✓	✓
5	ACTB	✓	✓
6	ANXA2	✓	✓
7	CD63		
8	SDCBP		
9	ENO1	✓	✓
10	HSP90AA1	✓	✓
11	TSG101		
12	PKM	✓	✓
13	LDHA	✓	✓
14	EEF1A1	✓	✓
15	YWHAZ	✓	✓
16	PGK1		✓
17	EEF2	✓	✓
18	ALDOA	✓	✓
19	HSP90AB1		✓
20	ANXA5	✓	✓
21	FASN		
22	YWHAE	✓	✓
23	CLTC	✓	
24	CD81	✓	✓
25	ALB		
26	VCP		
27	TPI1		✓
28	PPIA	✓	✓
29	MSN	✓	✓
30	CFL1	✓	✓
31	PRDX1	✓	✓
32	PFN1	✓	✓
33	RAP1B		
34	ITGB1	✓	✓
35	HSPA5		✓
36	SLC3A2	✓	✓
37	HIST1H4A		
38	GNB2	✓	✓
39	ATP1A1	✓	✓

40	YWHAQ		✓
41	FLOT1		
42	FLNA	✓	
43	CLIC1	✓	✓
44	CDC42		
45	CCT2		
46	A2M	✓	✓
47	YWHAG	✓	
48	TUBA1B		
49	RAC1		
50	LGALS3BP		
