# Supplementary material for: Exosomes Released by Corneal Stromal Cells Show Molecular Alterations in Keratoconus Patients and Induce Different Cellular Behavior
Source: Biomedicines. 2022 Sep 21;10(10):2348. doi: 10.3390/biomedicines10102348 (PMC9598276; doi:10.3390/biomedicines10102348)
Supplement: Supplementary file 1 [file biomedicines-10-02348-s001.zip › biomedicines-1778042-supplementary/Supplementary Table S3.pdf]

Supplementary Table S3

List of miRNAs identified by Next-Generation Sequencing in exosomes isolated from healthy stromal cell cultures and in isolates from keratoconus stromal cell cultures.

miRNA Name	Healthy stroma cells	KC Stroma cells
hsa-miR-3960	✓	✓
hsa-miR-4508	✓	✓
hsa-miR-3135b	✓	✓
hsa-miR-4516	✓	✓
hsa-miR-10400-5p	✓	✓
hsa-miR-4492	✓	✓
hsa-miR-3195	✓	✓
hsa-miR-10396b-5p	✓	
hsa-miR-10396a-5p	✓	
hsa-miR-146a-5p	✓	✓
hsa-miR-378a-5p	✓	✓
hsa-miR-4497	✓	✓
hsa-miR-7704	✓	✓
hsa-miR-598-3p	✓	✓
hsa-miR-23a-5p	✓	✓
hsa-miR-548j-5p	✓	✓
hsa-miR-663a	✓	
hsa-miR-1260b	✓	✓
hsa-miR-184	✓	✓
hsa-miR-335-5p	✓	✓
hsa-miR-4485-3p	✓	✓
hsa-miR-148a-3p	✓	✓
hsa-miR-4488	✓	✓
hsa-miR-34a-3p	✓	✓
hsa-miR-10401-3p	✓	
hsa-miR-369-5p	✓	✓
hsa-miR-3138	✓	
hsa-miR-378a-3p	✓	✓
hsa-miR-19a-3p	✓	✓
hsa-miR-219a-5p	✓	✓
hsa-miR-10396a-3p	✓	
hsa-miR-455-3p	✓	✓
hsa-miR-549a-3p	✓	✓
hsa-miR-3129-5p	✓	✓
hsa-miR-3117-3p	✓	✓
hsa-miR-3622a-5p	✓	✓
hsa-miR-3200-3p	✓	✓
hsa-miR-183-5p	✓	✓
hsa-miR-1306-3p	✓	
hsa-miR-223-5p	✓	
hsa-miR-181d-5p	✓	✓
hsa-miR-320e	✓	
hsa-miR-494-3p	✓	✓
hsa-miR-6724-5p	✓	✓
hsa-miR-27b-5p	✓	✓
hsa-miR-485-3p	✓	✓
hsa-let-7i-3p	✓	✓
hsa-miR-139-5p	✓	
hsa-miR-431-3p	✓	✓
hsa-miR-3648	✓	✓
hsa-miR-495-3p		✓
hsa-miR-548ad-5p		✓
hsa-miR-2355-3p	✓	✓
hsa-miR-548n	✓	
hsa-miR-3129-3p	✓	✓
hsa-miR-362-5p	✓	✓
hsa-miR-656-3p	✓	
hsa-miR-330-5p	✓	✓
hsa-miR-485-5p	✓	✓
hsa-miR-1301-3p	✓	✓
hsa-miR-205-5p	✓	✓
hsa-miR-708-3p	✓	✓
hsa-miR-3173-5p	✓	✓
hsa-miR-30a-5p	✓	✓
hsa-miR-133a-3p	✓	✓
hsa-miR-20a-3p	✓	
hsa-miR-6734-5p	✓	
hsa-miR-6745	✓	
hsa-miR-5697	✓	
hsa-miR-4463	✓	
hsa-miR-4636	✓	
hsa-miR-548c-5p	✓	
hsa-miR-6529-5p	✓	✓
hsa-miR-138-5p	✓	✓
hsa-miR-154-5p	✓	✓
hsa-miR-380-3p		✓
hsa-miR-3192-5p	✓	
hsa-miR-21-3p	✓	✓

hsa-miR-4676-3p		✓
hsa-miR-409-5p	✓	✓
hsa-miR-376c-3p	✓	✓
hsa-miR-6715a-3p	✓	✓
hsa-miR-2277-5p		✓
hsa-miR-137-3p	✓	✓
hsa-miR-4665-5p	✓	
hsa-miR-10527-5p	✓	
hsa-miR-193a-3p		✓
hsa-miR-1262	✓	✓
hsa-miR-26b-3p	✓	
hsa-miR-10a-5p	✓	✓
hsa-miR-125b-2-3p	✓	✓
hsa-miR-122-5p	✓	✓
hsa-miR-3661	✓	✓
hsa-miR-874-3p	✓	✓
hsa-miR-3928-3p	✓	✓
hsa-miR-296-3p	✓	✓
hsa-miR-101-3p	✓	✓
hsa-miR-505-3p	✓	✓
hsa-miR-181a-3p	✓	✓
hsa-miR-1260a	✓	✓
hsa-miR-5585-3p	✓	✓
hsa-miR-181b-3p	✓	✓
hsa-miR-12136	✓	✓
hsa-miR-320d	✓	✓
hsa-miR-1185-1-3p	✓	✓
hsa-miR-543	✓	✓
hsa-miR-4484		✓
hsa-miR-11181-3p		✓
hsa-miR-3196		✓
hsa-miR-6764-5p		✓
hsa-miR-5699-3p		✓
hsa-miR-3180-3p		✓
hsa-miR-6132		✓
hsa-miR-1910-5p		✓
hsa-miR-7851-3p		✓
hsa-miR-151b		✓
hsa-miR-5009-5p		✓
hsa-miR-449a		✓
hsa-miR-2276-3p		✓
hsa-miR-196a-5p		✓
hsa-miR-29b-2-5p		✓
hsa-miR-4763-3p		✓
hsa-miR-6851-5p		✓

hsa-miR-328-3p	✓	✓
hsa-miR-432-3p		✓
hsa-miR-370-3p	✓	✓
hsa-miR-549a-5p	✓	✓
hsa-miR-1303	✓	✓
hsa-miR-487b-3p	✓	✓
hsa-miR-329-3p	✓	✓
hsa-miR-1179	✓	✓
hsa-miR-302a-5p	✓	✓
hsa-miR-148a-5p	✓	✓
hsa-miR-197-3p	✓	✓
hsa-miR-212-5p	✓	✓
hsa-miR-758-5p	✓	✓
hsa-miR-548l		✓
hsa-miR-4768-5p		✓
hsa-miR-188-5p		✓
hsa-miR-6891-5p		✓
hsa-miR-320c	✓	✓
hsa-miR-4683		✓
hsa-miR-4659a-3p		✓
hsa-miR-144-5p	✓	✓
hsa-miR-9851-3p		✓
hsa-miR-5699-5p	✓	
hsa-miR-3131	✓	
hsa-miR-758-3p	✓	
hsa-miR-4688	✓	
hsa-miR-6859-5p	✓	
hsa-miR-374b-3p	✓	
hsa-miR-301b-3p	✓	
hsa-miR-219a-1-3p	✓	
hsa-miR-3158-3p	✓	
hsa-miR-539-3p	✓	
hsa-miR-6852-5p	✓	
hsa-miR-4767	✓	
hsa-miR-496	✓	
hsa-miR-6787-3p	✓	
hsa-miR-624-5p	✓	
hsa-miR-141-3p	✓	
hsa-miR-3133	✓	
hsa-miR-6892-5p	✓	
hsa-miR-380-5p	✓	✓
hsa-miR-665	✓	✓
hsa-miR-3154	✓	✓
hsa-miR-382-5p	✓	✓
hsa-miR-935	✓	✓

hsa-miR-1843	✓	✓
hsa-miR-383-5p	✓	
hsa-miR-144-3p	✓	✓
hsa-miR-490-3p	✓	
hsa-miR-490-5p	✓	
hsa-miR-6889-3p	✓	
hsa-miR-874-5p	✓	
hsa-miR-216a-5p	✓	
hsa-miR-302d-3p	✓	
hsa-miR-940	✓	
hsa-miR-3198	✓	✓
hsa-miR-433-3p	✓	
hsa-miR-4769-5p	✓	
hsa-miR-1291	✓	✓
hsa-miR-4677-3p	✓	✓
hsa-miR-99b-5p	✓	✓
hsa-miR-381-3p	✓	✓
hsa-miR-10a-3p	✓	✓
hsa-miR-142-3p	✓	✓
hsa-miR-150-5p	✓	✓
hsa-miR-6747-3p	✓	✓
hsa-miR-199a-5p	✓	✓
hsa-miR-34c-3p	✓	✓
hsa-miR-4421	✓	✓
hsa-miR-4466	✓	✓
hsa-miR-331-5p	✓	
hsa-miR-654-3p	✓	✓
hsa-miR-493-3p	✓	✓
hsa-miR-335-3p	✓	✓
hsa-miR-377-3p	✓	✓
hsa-miR-455-5p	✓	✓
hsa-miR-34b-5p	✓	✓
hsa-miR-1307-3p	✓	✓
hsa-miR-181c-5p	✓	✓
hsa-miR-877-5p	✓	✓
hsa-miR-450a-1-3p	✓	✓
hsa-miR-382-3p	✓	✓
hsa-miR-132-5p	✓	✓
hsa-miR-1468-5p	✓	✓
hsa-miR-203a-3p	✓	✓
hsa-miR-9-5p	✓	✓
hsa-miR-15b-3p	✓	✓
hsa-miR-1246	✓	✓
hsa-miR-4728-3p	✓	✓
hsa-miR-125a-5p	✓	✓

hsa-miR-3605-3p	✓	✓
hsa-miR-181a-5p	✓	✓
hsa-miR-1908-5p	✓	✓
hsa-miR-2277-3p	✓	✓
hsa-miR-1292-5p	✓	✓
hsa-miR-452-5p	✓	✓
hsa-miR-182-5p	✓	✓
hsa-miR-135b-5p	✓	✓
hsa-miR-7-5p	✓	✓
hsa-miR-616-3p	✓	✓
hsa-miR-5001-3p	✓	✓
hsa-miR-3182	✓	✓
hsa-miR-210-3p	✓	✓
hsa-miR-217-5p	✓	✓
hsa-miR-584-5p	✓	✓
hsa-miR-7-1-3p	✓	✓
hsa-miR-155-5p	✓	✓
hsa-miR-642a-5p	✓	✓
hsa-miR-2682-5p	✓	✓
hsa-miR-744-5p	✓	✓
hsa-miR-1273c	✓	✓
hsa-miR-423-5p	✓	✓
hsa-miR-375-3p	✓	✓
hsa-miR-409-3p	✓	✓
hsa-miR-31-5p	✓	✓
hsa-miR-502-3p	✓	✓
hsa-miR-589-5p	✓	✓
hsa-miR-3126-3p	✓	✓
hsa-miR-301a-3p	✓	✓
hsa-miR-218-5p	✓	✓
hsa-miR-146b-3p	✓	✓
hsa-miR-6500-3p	✓	✓
hsa-miR-127-5p	✓	✓
hsa-miR-323a-3p	✓	✓
hsa-miR-532-3p	✓	✓
hsa-miR-628-3p	✓	✓
hsa-miR-199a-3p	✓	✓
hsa-miR-181a-2-3p	✓	✓
hsa-miR-1304-3p	✓	✓
hsa-miR-25-3p	✓	✓
hsa-miR-1307-5p	✓	✓
hsa-miR-145-3p	✓	✓
hsa-miR-208b-3p		✓
hsa-miR-3940-3p		✓
hsa-miR-3151-5p		✓

hsa-miR-6781-5p		✓
hsa-miR-3150a-3p		✓
hsa-miR-3156-5p		✓
hsa-miR-4664-3p		✓
hsa-miR-7705		✓
hsa-miR-1180-5p		✓
hsa-miR-3913-3p		✓
hsa-miR-4762-3p		✓
hsa-miR-30d-3p		✓
hsa-miR-3620-3p		✓
hsa-miR-6131		✓
hsa-miR-187-3p		✓
hsa-miR-4504		✓
hsa-miR-3684		✓
hsa-miR-204-5p		✓
hsa-miR-548aw		✓
hsa-miR-541-5p		✓
hsa-miR-4717-3p		✓
hsa-miR-3691-3p		✓
hsa-miR-659-5p		✓
hsa-miR-149-3p		✓
hsa-miR-10395-3p		✓
hsa-miR-12135		✓
hsa-miR-497-3p		✓
hsa-miR-32-5p	✓	✓
hsa-miR-3617-5p		✓
hsa-miR-6847-5p		✓
hsa-miR-6855-3p		✓
hsa-miR-6835-5p		✓
hsa-miR-655-3p		✓
hsa-miR-4799-5p		✓
hsa-miR-6739-3p		✓
hsa-miR-7-2-3p		✓
hsa-miR-4753-5p		✓
hsa-miR-15a-3p		✓
hsa-miR-3675-3p		✓
hsa-miR-6791-5p		✓
hsa-miR-585-5p		✓
hsa-miR-519c-5p		✓
hsa-miR-6511b-5p		✓
hsa-miR-6779-5p		✓
hsa-miR-744-3p		✓
hsa-miR-6796-3p		✓
hsa-miR-3680-5p		✓
hsa-miR-4787-3p		✓

hsa-miR-500b-3p		✓
hsa-miR-376a-2-5p		✓
hsa-miR-552-5p		✓
hsa-miR-3121-3p		✓
hsa-miR-3912-3p		✓
hsa-miR-1277-3p		✓
hsa-miR-3688-3p		✓
hsa-miR-1323		✓
hsa-miR-3127-3p		✓
hsa-miR-7155-5p		✓
hsa-miR-3605-5p		✓
hsa-miR-6875-5p		✓
hsa-miR-3934-3p		✓
hsa-miR-139-3p		✓
hsa-miR-765		✓
hsa-miR-6733-5p		✓
hsa-miR-7111-5p		✓
hsa-miR-6820-3p		✓
hsa-miR-3152-3p		✓
hsa-miR-3941		✓
hsa-miR-95-3p		✓
hsa-miR-6728-5p		✓
hsa-miR-6513-5p		✓
hsa-miR-4729		✓
hsa-miR-6767-5p		✓
hsa-miR-4467		✓
hsa-miR-6825-5p		✓
hsa-miR-5701		✓
hsa-miR-6873-3p		✓
hsa-miR-1343-3p	✓	✓
hsa-miR-1305		✓
hsa-miR-3187-3p	✓	✓
hsa-let-7c-5p	✓	✓
hsa-miR-134-5p	✓	✓
hsa-miR-3940-5p	✓	✓
hsa-miR-3180	✓	✓
hsa-miR-2682-3p	✓	✓
hsa-miR-361-3p	✓	✓
hsa-miR-92a-3p	✓	✓
hsa-miR-1185-5p	✓	✓
hsa-miR-501-3p	✓	✓
hsa-miR-25-5p	✓	✓
hsa-miR-1285-5p	✓	✓
hsa-miR-181b-5p	✓	✓
hsa-miR-30c-5p	✓	✓

hsa-miR-29c-3p	✓	✓
hsa-miR-126-3p	✓	✓
hsa-miR-6799-5p	✓	
hsa-miR-6869-5p	✓	
hsa-miR-3159	✓	
hsa-miR-6751-5p	✓	
hsa-miR-1343-5p	✓	
hsa-miR-203b-3p	✓	
hsa-miR-363-3p	✓	
hsa-miR-6742-3p	✓	
hsa-miR-616-5p	✓	
hsa-miR-378d	✓	
hsa-miR-4668-5p	✓	
hsa-miR-4726-5p	✓	
hsa-miR-4766-3p	✓	
hsa-miR-1261	✓	
hsa-miR-652-5p	✓	
hsa-miR-141-5p	✓	
hsa-miR-194-3p	✓	
hsa-miR-1915-3p	✓	
hsa-miR-6837-3p	✓	
hsa-miR-3616-3p	✓	
hsa-miR-3170	✓	
hsa-miR-602	✓	
hsa-miR-4734	✓	
hsa-miR-204-3p	✓	
hsa-miR-376c-5p	✓	
hsa-miR-4803	✓	
hsa-miR-6757-5p	✓	
hsa-miR-6826-5p	✓	
hsa-miR-3622a-3p	✓	
hsa-miR-6777-3p	✓	
hsa-miR-4797-3p	✓	
hsa-miR-4524a-3p	✓	
hsa-miR-24-2-5p	✓	
hsa-miR-4669	✓	
hsa-miR-582-5p	✓	
hsa-miR-5579-3p	✓	
hsa-miR-4677-5p	✓	
hsa-miR-654-5p	✓	
hsa-miR-597-3p	✓	
hsa-miR-3909	✓	
hsa-miR-4788	✓	
hsa-miR-3124-5p	✓	
hsa-miR-4639-5p	✓	

hsa-miR-588	✓	
hsa-miR-450b-3p	✓	
hsa-miR-4482-3p	✓	
hsa-miR-548w	✓	
hsa-miR-618	✓	
hsa-miR-4747-3p	✓	
hsa-miR-4716-3p	✓	
hsa-miR-410-3p	✓	
hsa-miR-487a-3p	✓	
hsa-miR-371a-5p	✓	
hsa-miR-1255a	✓	
hsa-miR-3167	✓	
hsa-miR-6877-5p	✓	
hsa-miR-5001-5p	✓	
hsa-miR-92a-1-5p	✓	
hsa-miR-3115	✓	
hsa-miR-4435	✓	
hsa-miR-6509-5p	✓	
hsa-miR-20b-5p	✓	
hsa-miR-4773	✓	
hsa-miR-6893-5p	✓	
hsa-miR-370-5p	✓	
hsa-miR-3146	✓	
hsa-miR-4685-5p	✓	
hsa-miR-1908-3p	✓	
hsa-miR-2116-3p	✓	
hsa-miR-4479	✓	
hsa-miR-9901	✓	
hsa-miR-4449	✓	
hsa-miR-7151-3p	✓	
hsa-miR-181b-2-3p	✓	
hsa-miR-6826-3p	✓	
hsa-miR-3161	✓	
hsa-miR-6736-5p	✓	
hsa-miR-5583-3p	✓	
hsa-miR-548q	✓	
hsa-miR-1914-3p	✓	
hsa-miR-3160-5p	✓	
hsa-miR-4710	✓	
hsa-miR-4658	✓	
hsa-miR-1276	✓	
hsa-miR-302a-3p	✓	
hsa-miR-433-5p	✓	
hsa-miR-1197	✓	
hsa-miR-3174	✓	

hsa-miR-545-3p	✓	
hsa-miR-5000-3p	✓	
hsa-miR-1237-3p	✓	
hsa-miR-627-3p	✓	
hsa-miR-5008-5p	✓	
hsa-miR-509-3p	✓	
hsa-miR-4999-5p	✓	
hsa-miR-4470	✓	
hsa-miR-4687-3p	✓	
hsa-miR-6785-5p	✓	
hsa-miR-6721-5p	✓	
hsa-miR-541-3p	✓	
hsa-miR-10396b-3p	✓	
hsa-miR-4783-3p	✓	
hsa-miR-6871-5p	✓	
hsa-miR-6828-3p	✓	
hsa-miR-6797-5p	✓	
hsa-miR-6730-5p	✓	
hsa-miR-199b-5p	✓	✓
hsa-miR-532-5p	✓	✓
hsa-miR-542-5p	✓	✓
hsa-miR-3127-5p	✓	✓
hsa-miR-574-5p	✓	✓
hsa-miR-451a	✓	✓
hsa-miR-376a-3p	✓	✓
hsa-miR-493-5p	✓	✓
hsa-miR-424-5p	✓	✓
hsa-miR-29a-5p	✓	✓
hsa-miR-431-5p	✓	✓
hsa-miR-301a-5p	✓	✓
hsa-miR-1270	✓	✓
hsa-miR-1255b-5p	✓	✓
hsa-miR-21-5p	✓	✓
hsa-miR-106b-5p	✓	✓
hsa-miR-195-5p	✓	✓
hsa-miR-222-5p	✓	✓
hsa-miR-450a-2-3p	✓	✓
hsa-miR-1-3p	✓	✓
hsa-miR-3164	✓	✓
hsa-miR-379-5p	✓	✓
hsa-miR-4804-5p	✓	✓
hsa-miR-342-5p	✓	✓
hsa-miR-642a-3p	✓	✓
hsa-let-7b-5p	✓	✓
hsa-miR-629-5p	✓	✓

hsa-miR-1226-3p	✓	✓
hsa-miR-215-5p	✓	✓
hsa-miR-1228-5p	✓	✓
hsa-miR-548b-5p	✓	✓
hsa-miR-629-3p	✓	✓
hsa-miR-206	✓	✓
hsa-miR-1297	✓	✓
hsa-miR-8485	✓	✓
hsa-miR-769-3p	✓	✓
hsa-miR-651-5p	✓	✓
hsa-miR-17-5p	✓	✓
hsa-miR-34c-5p	✓	✓
hsa-miR-22-5p	✓	✓
hsa-miR-450a-5p	✓	✓
hsa-miR-4748	✓	✓
hsa-miR-9985	✓	✓
hsa-miR-937-5p	✓	✓
hsa-miR-652-3p	✓	✓
hsa-miR-19b-3p	✓	✓
hsa-miR-214-5p	✓	✓
hsa-miR-660-5p	✓	✓
hsa-miR-561-5p	✓	✓
hsa-miR-221-5p	✓	✓
hsa-miR-4286	✓	✓
hsa-miR-92b-3p	✓	✓
hsa-miR-374a-3p	✓	✓
hsa-miR-299-3p	✓	✓
hsa-miR-641	✓	✓
hsa-miR-125a-3p	✓	✓
hsa-miR-885-5p	✓	✓
hsa-miR-192-5p	✓	✓
hsa-miR-6827-3p	✓	✓
hsa-miR-148b-5p	✓	✓
hsa-miR-34a-5p	✓	✓
hsa-miR-193a-5p	✓	✓
hsa-let-7a-2-3p	✓	✓
hsa-miR-4454	✓	✓
hsa-miR-324-3p	✓	✓
hsa-miR-224-5p	✓	✓
hsa-miR-99a-5p	✓	✓
hsa-miR-146b-5p	✓	✓
hsa-miR-4510	✓	✓
hsa-miR-376b-5p	✓	✓
hsa-miR-501-5p	✓	✓
hsa-miR-376b-3p	✓	✓

hsa-miR-10401-5p	✓	✓
hsa-miR-6769b-3p	✓	✓
hsa-miR-6716-3p	✓	✓
hsa-miR-6514-5p	✓	✓
hsa-miR-3175	✓	✓
hsa-miR-338-3p	✓	✓
hsa-miR-5698	✓	✓
hsa-miR-3157-5p	✓	✓
hsa-miR-494-5p	✓	✓
hsa-miR-379-3p	✓	✓
hsa-miR-664b-5p	✓	✓
hsa-miR-4791	✓	✓
hsa-miR-200b-5p	✓	✓
hsa-miR-4443	✓	✓
hsa-miR-548k	✓	✓
hsa-miR-99a-3p	✓	✓
hsa-miR-589-3p	✓	✓
hsa-miR-193b-5p	✓	✓
hsa-miR-497-5p	✓	✓
hsa-miR-628-5p	✓	✓
hsa-miR-320b	✓	✓
hsa-miR-1285-3p	✓	✓
hsa-miR-545-5p	✓	✓
hsa-miR-708-5p	✓	✓
hsa-miR-143-5p	✓	✓
hsa-miR-143-3p	✓	✓
hsa-miR-3176	✓	✓
hsa-miR-30c-2-3p	✓	✓
hsa-miR-671-3p	✓	✓
hsa-miR-643	✓	✓
hsa-miR-1306-5p	✓	✓
hsa-miR-136-3p	✓	✓
hsa-miR-28-5p	✓	✓
hsa-miR-98-5p	✓	✓
hsa-miR-200b-3p	✓	✓
hsa-miR-548h-5p	✓	✓
hsa-miR-15a-5p	✓	✓
hsa-miR-27a-3p	✓	✓
hsa-miR-450b-5p	✓	✓
hsa-miR-10526-3p	✓	✓
hsa-miR-503-5p	✓	✓
hsa-miR-576-5p	✓	✓
hsa-miR-2278	✓	✓
hsa-miR-1273h-5p	✓	✓
hsa-miR-454-3p	✓	✓
hsa-miR-96-5p	✓	✓
hsa-miR-29a-3p	✓	✓
hsa-miR-147b-3p	✓	✓
hsa-miR-340-5p	✓	✓
hsa-miR-151a-5p	✓	✓
hsa-miR-454-5p	✓	✓
hsa-miR-200c-3p	✓	✓
hsa-miR-142-5p	✓	✓
hsa-miR-126-5p	✓	✓
hsa-miR-7706	✓	✓
hsa-miR-31-3p	✓	✓
hsa-miR-185-5p	✓	✓
hsa-miR-4741	✓	✓
hsa-miR-887-3p	✓	✓
hsa-miR-190b-5p	✓	✓
hsa-miR-93-5p	✓	✓
hsa-miR-1293	✓	✓
hsa-miR-138-1-3p	✓	✓
hsa-miR-128-1-5p	✓	✓
hsa-miR-671-5p	✓	✓
hsa-miR-3679-5p	✓	✓
hsa-miR-889-3p	✓	✓
hsa-miR-29c-5p	✓	✓
hsa-miR-339-3p	✓	✓
hsa-miR-1287-5p	✓	✓
hsa-miR-3934-5p	✓	✓
hsa-miR-411-5p	✓	✓
hsa-miR-766-3p	✓	✓
hsa-miR-326	✓	✓
hsa-miR-26a-2-3p	✓	✓
hsa-miR-365a-5p	✓	✓
hsa-miR-590-3p	✓	✓
hsa-miR-574-3p	✓	✓
hsa-miR-500a-3p	✓	✓
hsa-miR-4429	✓	✓
hsa-miR-1290	✓	✓
hsa-miR-222-3p	✓	✓
hsa-miR-10b-5p	✓	✓
hsa-miR-30a-3p	✓	✓
hsa-miR-1275	✓	✓
hsa-miR-491-5p	✓	✓
hsa-miR-30d-5p	✓	✓
hsa-miR-16-2-3p	✓	✓
hsa-miR-342-3p	✓	✓
hsa-miR-423-3p	✓	✓

hsa-miR-99b-3p	✓	✓
hsa-miR-625-3p	✓	✓
hsa-miR-23b-5p	✓	✓
hsa-let-7a-3p	✓	✓
hsa-miR-365a-3p	✓	✓
hsa-miR-130b-5p	✓	✓
hsa-miR-92b-5p	✓	✓
hsa-miR-7977	✓	✓
hsa-let-7i-5p	✓	✓
hsa-miR-17-3p	✓	✓
hsa-miR-26b-5p	✓	✓
hsa-miR-103a-3p	✓	✓
hsa-miR-374a-5p	✓	✓
hsa-miR-127-3p	✓	✓
hsa-miR-129-5p	✓	✓
hsa-miR-140-3p	✓	✓
hsa-miR-503-3p	✓	✓
hsa-miR-365b-5p	✓	✓
hsa-miR-323b-3p	✓	✓
hsa-miR-345-3p	✓	✓
hsa-miR-432-5p	✓	✓
hsa-miR-296-5p	✓	✓
hsa-miR-130a-3p	✓	✓
hsa-miR-221-3p	✓	✓
hsa-miR-186-5p	✓	✓
hsa-let-7d-5p	✓	✓
hsa-miR-122b-5p	✓	✓
hsa-miR-181c-3p	✓	✓
hsa-let-7a-5p	✓	✓
hsa-miR-149-5p	✓	✓
hsa-miR-339-5p	✓	✓
hsa-miR-18a-3p	✓	✓
hsa-miR-942-5p	✓	✓
hsa-miR-331-3p	✓	✓
hsa-miR-330-3p	✓	✓
hsa-miR-34b-3p	✓	✓
hsa-miR-106b-3p	✓	✓
hsa-miR-1268a	✓	✓
hsa-miR-27b-3p	✓	✓
hsa-miR-191-3p	✓	✓
hsa-miR-412-5p	✓	✓
hsa-miR-22-3p	✓	✓
hsa-let-7g-3p	✓	✓
hsa-miR-223-3p	✓	✓
hsa-miR-1294	✓	✓

hsa-miR-137-5p	✓	✓
hsa-miR-4725-3p	✓	✓
hsa-miR-145-5p	✓	✓
hsa-miR-664a-5p	✓	✓
hsa-miR-190a-5p	✓	✓
hsa-miR-619-5p	✓	✓
hsa-miR-1296-5p	✓	✓
hsa-miR-2110	✓	✓
hsa-miR-28-3p	✓	✓
hsa-miR-424-3p	✓	✓
hsa-miR-1271-5p	✓	✓
hsa-miR-193b-3p	✓	✓
hsa-miR-345-5p	✓	✓
hsa-miR-26a-5p	✓	✓
hsa-miR-3615	✓	✓
hsa-miR-16-5p	✓	✓
hsa-miR-27a-5p	✓	✓
hsa-miR-148b-3p	✓	✓
hsa-miR-337-5p	✓	✓
hsa-miR-107	✓	✓
hsa-miR-3613-5p	✓	✓
hsa-let-7e-5p	✓	✓
hsa-miR-185-3p	✓	✓
hsa-miR-769-5p	✓	✓
hsa-miR-214-3p	✓	✓
hsa-miR-1299	✓	✓
hsa-miR-30e-3p	✓	✓
hsa-miR-664a-3p	✓	✓
hsa-miR-29b-3p	✓	✓
hsa-miR-941	✓	✓
hsa-miR-30e-5p	✓	✓
hsa-miR-3065-3p	✓	✓
hsa-miR-125b-5p	✓	✓
hsa-miR-140-5p	✓	✓
hsa-miR-125b-1-3p	✓	✓
hsa-miR-18a-5p	✓	✓
hsa-miR-760	✓	✓
hsa-miR-425-5p	✓	✓
hsa-miR-625-5p	✓	✓
hsa-miR-151a-3p	✓	✓
hsa-let-7f-5p	✓	✓
hsa-miR-376a-5p	✓	✓
hsa-miR-320a-3p	✓	✓
hsa-miR-361-5p	✓	✓
hsa-let-7g-5p	✓	✓

hsa-miR-132-3p	✓	✓
hsa-let-7b-3p	✓	✓
hsa-miR-29b-1-5p	✓	✓
hsa-miR-15b-5p	✓	✓
hsa-miR-548d-5p	✓	✓
hsa-miR-299-5p	✓	✓
hsa-miR-128-3p	✓	✓
hsa-miR-449c-5p	✓	✓
hsa-miR-500a-5p	✓	✓
hsa-let-7e-3p	✓	✓
hsa-miR-302b-3p	✓	✓
hsa-miR-3944-3p	✓	✓
hsa-miR-329-5p	✓	✓
hsa-miR-200a-3p	✓	✓
hsa-miR-5187-5p	✓	✓
hsa-miR-582-3p	✓	✓
hsa-miR-197-5p	✓	✓
hsa-miR-195-3p	✓	✓
hsa-miR-1268b	✓	✓
hsa-miR-9-3p	✓	✓
hsa-miR-337-3p	✓	✓
hsa-miR-6821-5p	✓	✓
hsa-miR-4785	✓	✓
hsa-miR-26a-1-3p	✓	✓
hsa-miR-597-5p	✓	✓
hsa-miR-627-5p	✓	✓
hsa-miR-4746-5p	✓	✓
hsa-miR-6511a-3p	✓	✓
hsa-miR-212-3p	✓	✓
hsa-miR-377-5p	✓	✓
hsa-miR-6780a-5p	✓	✓
hsa-miR-576-3p	✓	✓
hsa-miR-362-3p	✓	✓
hsa-miR-106a-5p	✓	✓
hsa-miR-6834-5p	✓	✓
hsa-miR-5189-5p	✓	✓
hsa-miR-4660	✓	✓
hsa-miR-4440	✓	✓
hsa-let-7f-2-3p	✓	✓
hsa-miR-548a-3p	✓	✓
hsa-miR-1915-5p	✓	✓
hsa-miR-4742-3p	✓	✓
hsa-miR-429	✓	✓
hsa-miR-487b-5p	✓	✓
hsa-miR-33a-5p	✓	✓

hsa-miR-6862-5p	✓	✓
hsa-miR-548h-3p	✓	✓
hsa-miR-4747-5p	✓	✓
hsa-miR-6511b-3p	✓	✓
hsa-miR-152-5p	✓	✓
hsa-miR-3141	✓	✓
hsa-miR-548au-5p	✓	✓
hsa-miR-486-5p	✓	✓
hsa-miR-1180-3p	✓	✓
hsa-miR-9903	✓	✓
hsa-miR-548ay-5p	✓	✓
hsa-miR-378i	✓	✓
hsa-miR-422a	✓	✓
hsa-miR-5587-5p	✓	✓
hsa-miR-1284	✓	✓
hsa-miR-7976	✓	✓
hsa-miR-6513-3p	✓	✓
hsa-miR-6720-5p	✓	✓
hsa-miR-3065-5p	✓	✓
hsa-miR-542-3p	✓	✓
hsa-miR-4448	✓	✓
hsa-miR-324-5p	✓	✓
hsa-miR-100-5p	✓	✓
hsa-miR-1236-5p	✓	✓
hsa-miR-3140-3p	✓	✓
hsa-miR-4775	✓	✓
hsa-miR-23b-3p	✓	✓
hsa-miR-23a-3p	✓	✓
hsa-miR-374b-5p	✓	✓
hsa-let-7f-1-3p	✓	✓
hsa-miR-20a-5p	✓	✓
hsa-miR-194-5p	✓	✓
hsa-let-7d-3p	✓	✓
hsa-miR-136-5p	✓	✓
hsa-miR-24-3p	✓	✓
hsa-miR-152-3p	✓	✓
hsa-miR-484	✓	✓
hsa-miR-93-3p	✓	✓
hsa-miR-505-5p	✓	✓
hsa-miR-369-3p	✓	✓
hsa-miR-421	✓	✓
hsa-miR-130b-3p	✓	✓
hsa-miR-425-3p	✓	✓
hsa-miR-191-5p	✓	✓
hsa-miR-98-3p	✓	✓

hsa-miR-100-3p	✓	✓
hsa-miR-30b-5p	✓	✓
TOTAL	**692**	**642**
