# Supplementary material for: Exosomes Released by Corneal Stromal Cells Show Molecular Alterations in Keratoconus Patients and Induce Different Cellular Behavior
Source: Biomedicines. 2022 Sep 21;10(10):2348. doi: 10.3390/biomedicines10102348 (PMC9598276; doi:10.3390/biomedicines10102348)
Supplement: Supplementary file 1 [file biomedicines-10-02348-s001.zip › biomedicines-1778042-supplementary/Supplementary Table S4.pdf]

Supplementary Table S4

List of miRNAs species that appeared consistently in all samples of the same type

	Average normalized reads	
miRNA	Healthy	keratoconus
**hsa-let-7a-5p**	179543,743	177037,651
**hsa-let-7b-5p**	156244,880	179822,492
**hsa-miR-199a-3p**	55200,691	64958,271
**hsa-let-7f-5p**	54886,380	48997,542
**hsa-miR-125b-5p**	50885,743	44903,732
**hsa-miR-29a-3p**	46663,843	50069,631
**hsa-let-7i-5p**	43747,035	45597,751
**hsa-miR-100-5p**	42684,505	40678,358
**hsa-miR-221-3p**	32274,264	26931,932
**hsa-miR-4516**	31490,307	1912,463
**hsa-miR-16-5p**	31286,311	30933,779
**hsa-miR-21-5p**	28713,377	34092,436
**hsa-miR-122-5p**	20244,370	32294,878
**hsa-let-7e-5p**	11091,458	9838,946
**hsa-miR-143-3p**	11042,222	8359,044
**hsa-miR-103a-3p**	9860,929	8164,551
**hsa-miR-23a-3p**	9572,209	8641,335
**hsa-miR-31-5p**	7860,106	5064,295
**hsa-miR-155-5p**	7536,047	10947,555
**hsa-miR-24-3p**	6935,124	6384,026
**hsa-miR-191-5p**	6883,099	6264,058
**hsa-miR-125a-5p**	6614,273	4168,764
**hsa-miR-26a-5p**	6109,304	5934,336
**hsa-miR-151a-3p**	6025,678	5818,905
**hsa-miR-34a-5p**	5154,276	5501,793
**hsa-miR-93-5p**	4422,899	4641,164
**hsa-miR-1246**	4342,480	6360,363
**hsa-miR-99b-5p**	4022,325	2818,843
**hsa-miR-27b-3p**	3893,550	3647,111
**hsa-miR-26b-5p**	3679,140	3621,807
**hsa-miR-146a-5p**	3470,265	16696,611
**hsa-miR-152-3p**	3254,322	2981,966
**hsa-miR-423-5p**	3245,776	3915,340
**hsa-miR-30a-5p**	3128,691	4504,792
**hsa-miR-30d-5p**	3084,225	3187,551
**hsa-miR-25-3p**	2799,787	3292,028
**hsa-miR-3960**	2774,801	27,000
**hsa-let-7c-5p**	2739,768	3064,364
**hsa-miR-3135b**	2488,077	87,391
**hsa-miR-23b-3p**	2435,851	2202,695
**hsa-miR-27a-3p**	2230,101	2260,703
**hsa-miR-425-5p**	1996,207	1882,615
**hsa-let-7g-5p**	1946,966	1739,831
**hsa-miR-1307-3p**	1852,910	1198,392
**hsa-miR-320a-3p**	1826,138	1707,842
**hsa-miR-12136**	1814,984	2337,575
**hsa-miR-222-3p**	1723,294	1728,730
**hsa-miR-125b-1-3p**	1716,724	1677,619
**hsa-miR-92a-3p**	1682,663	1906,588
**hsa-miR-29b-3p**	1679,548	1592,225
**hsa-miR-30e-5p**	1552,627	1486,350
**hsa-miR-22-3p**	1465,069	1193,231
**hsa-let-7d-5p**	1372,670	1169,491
**hsa-miR-218-5p**	1348,357	769,669
**hsa-miR-432-5p**	1310,269	1084,481
**hsa-miR-193a-5p**	1306,368	1538,590
**hsa-miR-342-3p**	1281,377	1081,021
**hsa-miR-126-3p**	1246,117	792,262
**hsa-miR-28-3p**	1234,945	1238,086
**hsa-miR-128-3p**	1139,978	1053,323
**hsa-miR-4488**	1135,553	388,095
**hsa-miR-382-5p**	1096,420	607,623
**hsa-miR-423-3p**	1068,868	941,186
**hsa-miR-7-5p**	1042,512	1479,499
**hsa-miR-186-5p**	1027,630	1078,653
**hsa-miR-148b-3p**	1008,410	855,489
**hsa-miR-409-3p**	990,059	606,444
**hsa-miR-486-5p**	984,033	880,224
**hsa-miR-127-3p**	951,644	783,990
**hsa-miR-574-5p**	883,283	1193,501
**hsa-miR-671-5p**	882,545	989,859
**hsa-miR-4508**	882,152	8,597
**hsa-miR-138-5p**	855,295	343,591
**hsa-miR-224-5p**	852,898	1035,527
**hsa-miR-29c-3p**	841,318	1101,319
**hsa-miR-4497**	829,917	217,678
**hsa-miR-134-5p**	826,423	510,987
**hsa-miR-148a-3p**	818,587	1963,804
**hsa-miR-361-5p**	808,278	741,841
**hsa-miR-320c**	805,464	1364,470

**hsa-miR-199a-5p**	803,968	1206,144
**hsa-miR-140-3p**	800,965	647,971
**hsa-miR-142-3p**	792,072	448,891
**hsa-miR-339-5p**	781,577	671,744
**hsa-miR-146b-5p**	747,200	827,029
**hsa-miR-98-5p**	719,326	560,558
**hsa-miR-10a-5p**	715,611	1309,040
**hsa-miR-379-5p**	686,988	474,765
**hsa-miR-181a-5p**	657,623	930,791
**hsa-miR-1290**	636,600	712,155
**hsa-miR-15b-5p**	620,755	567,025
**hsa-miR-365a-3p**	609,060	476,205
**hsa-miR-199b-5p**	590,802	720,871
**hsa-miR-320d**	542,304	942,203
**hsa-miR-574-3p**	540,173	580,357
**hsa-miR-221-5p**	517,655	373,656
**hsa-miR-185-5p**	508,737	410,676
**hsa-miR-744-5p**	498,901	319,548
**hsa-miR-106b-3p**	476,307	414,204
**hsa-miR-454-3p**	474,712	386,331
**hsa-miR-708-5p**	466,639	522,991
**hsa-miR-137-3p**	437,835	222,017
**hsa-miR-361-3p**	422,728	560,809
**hsa-miR-654-3p**	386,261	217,244
**hsa-miR-190a-5p**	382,943	337,424
**hsa-miR-181b-5p**	364,835	481,334
**hsa-miR-214-3p**	339,335	310,554
**hsa-miR-99b-3p**	325,710	259,217
**hsa-miR-10400-5p**	319,067	8,068
**hsa-miR-4492**	305,392	14,490
**hsa-miR-3195**	294,597	33,392
**hsa-miR-181a-2-3p**	286,738	369,725
**hsa-miR-503-5p**	279,303	286,691
**hsa-miR-20a-5p**	271,847	239,831
**hsa-miR-99a-5p**	267,526	279,440
**hsa-miR-370-3p**	260,337	129,473
**hsa-miR-625-3p**	255,511	298,904
**hsa-miR-145-5p**	253,112	233,363
**hsa-miR-19b-3p**	250,169	279,374
**hsa-miR-30a-3p**	248,659	302,820
**hsa-miR-769-5p**	246,800	266,680
**hsa-miR-532-5p**	243,544	266,438
**hsa-miR-223-3p**	237,677	70,379
**hsa-miR-30c-5p**	228,334	284,525

**hsa-miR-328-3p**	226,989	120,904
**hsa-miR-193b-5p**	223,196	213,329
**hsa-miR-411-5p**	218,269	171,053
**hsa-miR-381-3p**	218,233	121,993
**hsa-miR-660-5p**	215,881	241,085
**hsa-miR-101-3p**	212,657	309,925
**hsa-miR-149-5p**	208,166	180,358
**hsa-miR-130a-3p**	203,478	205,714
**hsa-miR-7704**	200,200	35,038
**hsa-miR-629-5p**	199,431	255,347
**hsa-miR-4448**	193,540	175,797
**hsa-miR-941**	191,841	209,365
**hsa-miR-143-5p**	180,551	144,552
**hsa-miR-181a-3p**	175,427	278,873
**hsa-miR-296-3p**	172,552	246,524
**hsa-miR-140-5p**	163,165	117,899
**hsa-miR-6529-5p**	154,520	252,048
**hsa-miR-126-5p**	152,419	120,676
**hsa-miR-431-5p**	151,743	110,025
**hsa-miR-151a-5p**	141,237	136,147
**hsa-miR-107**	139,674	33,921
**hsa-miR-1260b**	139,044	49,151
**hsa-miR-15a-5p**	133,650	119,847
**hsa-miR-365b-5p**	132,904	173,195
**hsa-miR-132-3p**	127,880	155,126
**hsa-miR-10526-3p**	127,006	89,312
**hsa-miR-589-5p**	124,779	71,739
**hsa-miR-671-3p**	121,063	0,000
**hsa-miR-1260a**	119,924	53,124
**hsa-miR-192-5p**	118,453	102,970
**hsa-miR-1287-5p**	117,400	75,291
**hsa-miR-197-3p**	113,245	40,872
**hsa-miR-584-5p**	112,053	61,571
**hsa-miR-493-3p**	110,591	75,319
**hsa-miR-494-3p**	110,464	51,266
**hsa-miR-3182**	109,229	52,444
**hsa-miR-4454**	108,236	83,510
**hsa-miR-374a-5p**	106,878	101,883
**hsa-miR-92b-5p**	106,578	102,002
**hsa-miR-1268a**	104,933	112,668
**hsa-miR-1-3p**	104,516	103,452
**hsa-miR-130b-3p**	103,724	98,090
**hsa-miR-1296-5p**	100,761	108,831
**hsa-miR-664a-5p**	99,669	86,683

**hsa-miR-92b-3p**	99,627	104,300
**hsa-miR-369-5p**	98,365	183,864
**hsa-miR-425-3p**	97,219	59,696
**hsa-miR-339-3p**	96,607	84,809
**hsa-miR-15b-3p**	95,946	43,819
**hsa-miR-34c-5p**	93,856	136,542
**hsa-miR-17-5p**	93,683	108,787
**hsa-miR-493-5p**	91,771	66,073
**hsa-miR-21-3p**	91,148	0,000
**hsa-miR-3615**	89,182	72,056
**hsa-miR-378a-3p**	87,705	108,575
**hsa-miR-4286**	84,289	49,890
**hsa-miR-345-5p**	83,359	95,084
**hsa-miR-30e-3p**	82,323	79,370
**hsa-miR-455-5p**	82,023	100,537
**hsa-miR-210-3p**	81,195	98,785
**hsa-miR-376c-3p**	80,853	33,800
**hsa-miR-500a-3p**	80,714	49,091
**hsa-miR-100-3p**	79,180	71,408
**hsa-miR-2682-3p**	78,469	59,802
**hsa-miR-25-5p**	76,783	42,730
**hsa-miR-335-5p**	76,334	165,725
**hsa-miR-576-5p**	75,772	75,380
**hsa-let-7d-3p**	74,690	61,313
**hsa-miR-22-5p**	73,979	41,461
**hsa-miR-324-3p**	73,798	103,483
**hsa-miR-31-3p**	73,706	56,948
**hsa-miR-1180-3p**	73,329	33,362
**hsa-miR-487b-3p**	72,420	143,177
**hsa-miR-324-5p**	71,964	77,209
**hsa-miR-193b-3p**	71,935	57,475
**hsa-miR-130b-5p**	70,539	86,575
**hsa-miR-150-5p**	68,834	28,148
**hsa-miR-376a-3p**	68,623	167,637
**hsa-miR-542-3p**	68,220	55,211
**hsa-miR-619-5p**	67,601	69,700
**hsa-miR-369-3p**	67,050	66,012
**hsa-miR-451a**	66,918	30,838
**hsa-miR-93-3p**	64,585	71,210
**hsa-miR-1307-5p**	64,076	86,516
**hsa-miR-491-5p**	61,795	51,116
**hsa-miR-1271-5p**	60,668	36,218
**hsa-let-7b-3p**	59,313	37,487
**hsa-miR-421**	59,168	58,897
**hsa-miR-1275**	56,305	52,308

**hsa-miR-424-5p**	56,124	68,373
**hsa-miR-484**	54,990	63,519
**hsa-miR-10b-5p**	54,759	46,384
**hsa-miR-127-5p**	53,462	23,495
**hsa-miR-1301-3p**	52,316	25,822
**hsa-miR-29b-1-5p**	52,143	47,700
**hsa-miR-760**	51,743	55,241
**hsa-miR-329-3p**	51,719	24,673
**hsa-miR-450b-5p**	51,454	48,381
**hsa-miR-138-1-3p**	50,778	40,931
**hsa-miR-222-5p**	49,527	60,694
**hsa-miR-337-5p**	49,492	37,925
**hsa-miR-769-3p**	48,954	83,387
**hsa-miR-1468-5p**	47,826	21,289
**hsa-miR-3679-5p**	46,992	44,556
**hsa-miR-485-5p**	46,083	15,517
**hsa-miR-1304-3p**	45,811	97,032
**hsa-miR-27a-5p**	45,122	49,181
**hsa-miR-323a-3p**	44,595	29,826
**hsa-miR-190b-5p**	43,768	56,298
**hsa-miR-4725-3p**	43,410	36,867
**hsa-miR-136-3p**	42,502	18,343
**hsa-miR-887-3p**	42,194	44,347
**hsa-miR-195-5p**	41,667	52,446
**hsa-miR-23b-5p**	41,575	28,679
**hsa-miR-7977**	40,575	24,326
**hsa-miR-505-3p**	40,221	75,063
**hsa-miR-345-3p**	40,048	40,583
**hsa-miR-194-5p**	39,874	25,324
**hsa-miR-154-5p**	38,659	15,457
**hsa-miR-330-5p**	37,993	9,806
**hsa-miR-34c-3p**	37,866	13,961
**hsa-miR-628-5p**	37,339	35,720
**hsa-miR-2110**	36,986	39,224
**hsa-miR-203a-3p**	36,512	49,032
**hsa-miR-182-5p**	36,512	10,894
**hsa-miR-183-5p**	35,904	3,445
**hsa-miR-320b**	35,770	66,284
**hsa-miR-3129-5p**	34,943	81,516
**hsa-miR-503-3p**	34,550	20,669
**hsa-miR-454-5p**	34,289	24,386
**hsa-miR-889-3p**	33,808	31,714
**hsa-miR-205-5p**	33,069	52,474
**hsa-miR-502-3p**	32,842	18,902
**hsa-miR-129-5p**	32,496	16,197

**hsa-miR-505-5p**	32,269	45,433
**hsa-let-7a-3p**	32,188	31,096
**hsa-miR-532-3p**	31,269	16,136
**hsa-miR-331-3p**	31,142	20,609
**hsa-miR-942-5p**	31,053	41,203
**hsa-miR-590-3p**	31,053	36,868
**hsa-miR-3187-3p**	31,007	21,878
**hsa-miR-455-3p**	30,961	75,502
**hsa-miR-501-3p**	30,834	43,817
**hsa-miR-185-3p**	30,788	19,552
**hsa-miR-29c-5p**	30,180	24,115
**hsa-miR-1306-5p**	30,007	25,293
**hsa-miR-296-5p**	29,526	36,988
**hsa-miR-142-5p**	28,034	23,117
**hsa-miR-382-3p**	27,518	12,722
**hsa-miR-125a-3p**	26,899	48,591
**hsa-miR-299-3p**	26,291	31,125
**hsa-miR-30c-2-3p**	26,164	41,339
**hsa-miR-200c-3p**	25,990	25,202
**hsa-miR-1270**	24,937	38,515
**hsa-miR-18a-5p**	24,763	20,851
**hsa-miR-3613-5p**	24,155	21,380
**hsa-miR-625-5p**	24,063	18,962
**hsa-miR-133a-3p**	23,236	43,998
**hsa-miR-3117-3p**	23,236	1,148
**hsa-miR-3065-5p**	22,836	26,970
**hsa-miR-29a-5p**	22,801	17,285
**hsa-miR-34b-5p**	22,582	5,213
**hsa-miR-5585-3p**	22,101	10,984
**hsa-miR-181c-3p**	22,055	17,753
**hsa-miR-340-5p**	21,928	7,510
**hsa-miR-132-5p**	21,620	50,118
**hsa-miR-424-3p**	21,447	14,339
**hsa-miR-409-5p**	21,447	7,449
**hsa-let-7a-2-3p**	21,228	13,810
**hsa-miR-3065-3p**	21,228	6,890
**hsa-miR-191-3p**	20,874	19,023
**hsa-miR-6724-5p**	20,047	5,213
**hsa-miR-1294**	19,874	9,217
**hsa-miR-497-5p**	19,566	26,442
**hsa-miR-874-3p**	19,347	28,919
**hsa-miR-326**	18,566	15,049
**hsa-miR-708-3p**	18,519	20,730
**hsa-miR-1299**	18,258	25,882
**hsa-miR-3127-5p**	17,993	12,072

**hsa-let-7e-3p**	17,865	8,129
**hsa-miR-122b-5p**	17,865	6,301
**hsa-miR-214-5p**	17,385	28,269
**hsa-miR-3928-3p**	17,385	6,301
**hsa-miR-323b-3p**	16,731	16,136
**hsa-miR-7706**	16,112	27,181
**hsa-miR-4775**	14,757	16,666
**hsa-miR-374a-3p**	14,676	18,433
**hsa-miR-301a-3p**	13,976	12,722
**hsa-miR-542-5p**	13,403	9,217
**hsa-miR-3605-3p**	13,403	8,068
**hsa-miR-23a-5p**	13,322	39,722
**hsa-miR-144-3p**	12,622	2,916
**hsa-miR-1293**	12,141	14,369
**hsa-miR-3154**	11,968	2,916
**hsa-miR-320e**	11,968	0,000
**hsa-miR-19a-3p**	11,487	55,013
**hsa-miR-548h-5p**	10,787	12,133
**hsa-miR-2682-5p**	10,614	4,004
**hsa-miR-642a-5p**	10,614	2,916
**hsa-miR-3192-5p**	10,614	0,000
**hsa-miR-1262**	9,433	34,978
**hsa-miR-1291**	8,606	13,220
**hsa-miR-549a-3p**	8,125	28,149
**hsa-miR-181d-5p**	7,425	35,869
**hsa-miR-299-5p**	6,771	13,220
**hsa-miR-548d-5p**	6,771	12,133
**hsa-miR-184**	6,725	36,806
**hsa-miR-34a-3p**	6,725	30,626
**hsa-miR-215-5p**	6,725	15,049
**hsa-miR-1303**	6,071	20,881
**hsa-miR-1843**	6,071	17,345
**hsa-miR-128-1-5p**	4,717	10,984
**hsa-miR-1285-5p**	4,016	10,984
**hsa-miR-7-1-3p**	3,362	9,217
**hsa-miR-877-5p**	2,708	10,365
**hsa-miR-378a-5p**	2,008	31,865
**hsa-miR-4485-3p**	2,008	21,289
**hsa-miR-219a-5p**	2,008	16,197
**hsa-miR-2355-3p**	1,354	14,988
**hsa-miR-4466**	1,354	8,068
