# Supplementary material for: Exosomes Released by Corneal Stromal Cells Show Molecular Alterations in Keratoconus Patients and Induce Different Cellular Behavior
Source: Biomedicines. 2022 Sep 21;10(10):2348. doi: 10.3390/biomedicines10102348 (PMC9598276; doi:10.3390/biomedicines10102348)
Supplement: Supplementary file 1 [file biomedicines-10-02348-s001.zip › biomedicines-1778042-supplementary/Supplementary Table S5.pdf]

Supplementary Table S5

Prediction of the biological targets of miRNAs whose expression appears altered in exosomes of corneal cells from patients with keratoconus. The genes whose symbol appears in black are regulated by only one of the miRNAs with altered expression, in blue genes regulated by 2 are shown, in orange by 3 and in red by 4 different miRNAs

Downregulated miRNA	Regulated Genes
**hsa-miR-3182**	*AKAP10, AMOTL2, ANK3, ANKRD50, ANKS1B, ANP32E, AP4S1, ARHGAP21, ARID5B, ARPC3, ATP2B1, ATRX, B3GALNT2, BCAS1, BCL2L13, BMPR2, BROX, BRWD1, BTG1, BTRC, C11orf58, C11orf87, C12orf4, C1orf21, C2orf48, C5orf63, C8orf33, CACNA2D2, CALD1, CALML4, CAP2, CARD8, CCDC13, CCDC6, CCL16, CCNA2, CD22, CDH4, CDK2AP1, CHD1, CHML, CHST11, CHST15, CLCN3, CLTC, COL19A1, CREBZF, CREG1, CSNK1A1, CTSS, CYBB, DDX4, DDX55, DDX6, DIO2, DIXDC1, DLG2, DLG3, DPP10, DPY30, DRG2, E2F3, EEF1E1, EGLN1, ELAVL3, EML4, EML5, EOGT, EPHB1, EXD2, FAM102B, FAM160B1, FAM206A, FAM32A, FBP2, FBXW11, FCGR1A, FCGR1B, GABPB2, GABRB3, GALNT15, GBX2, GDA, GHITM, GLI2, GLIPR1, GMCL1, GNA14, GNPTAB, GPR158, GPRIN1, GRIK2, HAVCR2, HEXIM1, HOOK3, IARS, IER5, IFI44, IFRD2, IGFBP7, IL6ST, INTS8, ITGB5, JPH3, KATNAL1, KDELR1, KIAA1671, KLHL14, KLHL2, LAMC1, LRP6, LRP8, LRTM2, LYRM9, MAGI3, MALT1, MAP1B, MARCH8, MARVELD3, MET, METTL21A, METTL8, MIER1, MRRF, MTAP, MTMR10, MTMR4, MTOR, MYOCD, MYSM1, NCALD, NCKAP5, NDFIP2, NEU3, NMNAT2, NOL4, NPR3, NR3C1, NRIP1, NUFIP2, NYAP1, OPCML, PABPN1, PATE1, PHF2, PIK3CG, PLEKHH1, PPP1R12A, PPP4R2, PRKACB, PRKG2, PROX2, PSMB11, PSMC6, PSTK, PTBP3, PTP4A1, PTPN12, PTPRT, PUM1, PUM2, PYROXD1, RAB14, RAB2A, RALGAPA2, RALGPS2, RAP2A, RARB, RBM12, RHOU, RIT2, RNF169, RNF217, RNF222, RNF41, S100PBP, S1PR3, SBF2, SCN1B, SEMA5A, SGTB, SH2D4B, SH3BGRL2, SH3TC2, SIPA1L2, SLC16A14, SLC25A36, SLC5A12, SLC9B1, SMG7, SMIM13, SNAPC1, SNRNP27, SNX13, SNX19, SORL1, SPATA6, SPRY4, SRGAP2, SRXN1, SSTR3, STARD7, STK32A, STK35, SUCO, SUMO1, SUPT20H, TBL1XR1, TECTB, TEX19, TMC7, TMEM135, TMEM168, TMX1, TNFAIP8L2, TNFSF10, TNRC6B, TRIQK, TSHZ3, TSLP, TTC39B, UBE2W, UBR5, UFM1, USP28, WDR3, WDR41, WDR48, YTHDC1, ZBTB20, ZC3H14, ZDHHC20, ZFP36L1, ZMYND8, ZNF148, ZNF385B, ZNF418, ZNF682, ZNF697, ZNF81, ZRANB3, ZW10*
**hsa-miR-183-5p**	*39326, ABAT, ABCB10, ABI2, ABRAXAS2, ACER2, ACVR2B, AGPAT5, AJAP1, AKAP12, ALAD, AMD1, AMIGO2, AMMECR1L, ANKMY2, ANKRD13C, AP3M1, ARFGAP2, ARHGAP21, ARHGEF18, ARHGEF37, ARPP19, ATF2, ATP10B, ATP13A3, ATP2B4, ATRN, AZIN1, B3GNT2, BACH2, BAZ1B, BBOF1, BIRC6, BNC2, BNIP3L, C16orf72, CACNA1E, CACNB4, CALCB, CCDC121, CCL18, CDH9, CDK5R1, CELF2, CEP170B, CEP97, CFL2, CHD2, CLCN3, CPM, CSMD1, CTDSPL, CTNNA2, DAAM1, DAGLA, DCX, DDHD1, DEPDC5, DGCR2, DGKH, DIP2A, DMXL1, DNALI1, DUSP10, DYNC1I1, EMSY, ENAH, EPHA4, EPHA7, ERBIN, EZR, FAM217B, FCHO2, FKBP1A, FLRT3, FNDC3B, FOXN2, FRMD6, FUT2, GCLM, GJA3, GNG5, GOLPH3, GPAM, GPCPD1, GPR34, GSE1, GTF2H1, GYPA, HCN1, HTR2A, ICA1, IDH2, IMPAD1, ING3, IPPK, IRS1, ITGB1, ITGB8, KBTBD6, KCND2, KCNJ14, KCNK10, KCNK2, KCNMB1, KDM2B, KIAA0355, KIF13A, KIF2A, KLHL24, KLHL28, KLRD1, L3MBTL3, LCK, LHFPL2, LRP6, MACROD2, MAL2, MAP3K13, MAP3K4, MAP7D2, MAPK4, MAPK8IP1, MBNL1, MEF2C, MFSD6, MINAR1, MRC2, MTERF2, MTMR6, NARS2, NCAM2, NCK2, NEFL, NFYC, NPAS3, NR3C1, NRG1, NRIP2, NTRK2, NUDT4, NUFIP2, OSBPL8, OTUD4, PAM, PCK2, PDCD4, PDCD6, PEX19, PFN2,* ***PHF6****, PHLDB2, PKD2, PKP4, PLCB4, PLEKHA3, PLEKHA5, PLPPR2, POLR1C, PPP2CA, PPP2CB, PPP2R2A, PPP2R5C, PRKACB, PSEN2, PSMA5, PTDSS1, PTPN4, RAB21, RAB8B, RABGAP1L, RALA, RALGDS, RBMS1, RCN2, REPS2, REV1, REXO5, RFX3, RHOB, RHOBTB1, RHPN2, RIMBP2, RNF138, ROBO2, RORA, RPS6KA3, RSBN1, SACS, SAMD4A, SCN3A, SCYL3, SEL1L, SERP1, SESN1, SH3D19, SLAIN1, SLC1A2, SLC35A1, SLITRK1, SLITRK3, SMCO4, SMPD3, SOBP, SOCS6, SPATS2, SPC25, SPRY3, SREK1IP1, SRSF2, STC1, STK38L, TAB3, TAOK1, TCF12, TCF7L2, TET1, TMED7, TMEM184C, TMEM59, TMEM70, TMPO, TMSB4X, TOMM70, TRAM1, TRIM27, TTC7B, UNC13B, USF3, VDAC1, XKR6, XPNPEP3, XPOT, ZBTB34, ZDHHC6, ZEB1, ZFPM2, ZFYVE26, ZMYM2, ZNF197, ZNF750, ZNF770*
**hsa-miR-3117-3p**	*AFTPH, ANO4, APOL6, ASNS, AZI2, C21orf91, CADPS, CD274, CDC42BPA, CHM, CLDN18, CNOT6, COLGALT2, CSRNP3, DIP2B, DYNLL2, EHMT1, EID1, ELF3, FAM222B, FAM227A, FAM76B, GLRA2, GPT2, HAVCR1, KHDRBS1, KIF5A, KRAS, LHX2, NAP1L3, NID1, PAPOLB, PRKAB2, PSAT1, RALA, SCUBE2, STAG2, SYNCRIP, TBC1D24, UBXN10, ZFP2, ZHX2, ZNF441, ZNF680, ZNF74*

**hsa-miR-6724-5p**	*AMMECR1L, AOC3, B3GNT3, CD44, CHTF8, CYP1B1, GABBR2, GALNT6, GNG12, ITGB4, MTRNR2L10, MTRNR2L3, NAA15, NMT1, PAG1, RBMXL3, RSF1, SEMA4F, SLC25A42, SLC7A6, ST3GAL3, TSLP, UBE2J1, USP12, ZNF93*
**hsa-miR-3192-5p**	*AAK1, ABHD2, ACVR1B, ADAM29, ADARB2, ALKBH8, ANK1, APLP1, ARHGEF39, ARRB1, ATP8A2, ATRN, AVL9, BTG1, BTNL8, C1orf105, CALN1, CAPN10, CBX5, CCDC149, CDK5R1, CLIC5, CLIP3, CLSTN1, CNTLN, COQ5, CPD, CPEB1, CTNND2, CXCL12, CYP8B1, DCAF8, DNAJC11, DNM1L, EFR3B, ESRRB, ETS1, EXD2, FBXL7, FBXW7, FGF11, FHDC1, FSHR, G6PC3, GAL3ST3, GAL3ST4, GATA2, GGT7, GIGYF2, GOLGA6L10, GOLGA6L4, GOLGA6L9, HCK, HEYL, HIPK2, HMBOX1, HOOK2, IGDCC4, INPP5K, IQSEC3, IRF2, KCNC1, KCNQ4, KCTD14, KIAA0556, KLF1, KLHDC8A, KLHL18, KSR2, LDLRAP1, LPCAT2, LRRC59, LSM10, LYN, MAPK8IP3, MDM4, MEN1, METTL21A, MPZL1, MT1E, MT1F, MT1M, MYO1D, MYPOP, MYRF, N4BP1, NAV2, NDUFC2-KCTD14, NID1, NLGN3, NMNAT2, NRG1, NSUN2, ORAI2, OSBPL3, P4HA3, PCGF3, PDE3A, PHLPP2, PIM2, PITPNM2, PLP1, PRELP, PRICKLE2, PSMB11, PTGIS, RBMS2, RNPEPL1, RRNAD1, S100A5, SAV1, SBK1, SCRT2, SENP5, SERPINA9, SH3PXD2A, SH3TC2, SHISA7, SLC7A14, SLC7A8, SLCO2A1, SOX14, SPINK7, SPOCK1, SPRED3, STK36, STRADB, STX1B, SUSD5, SYNGAP1, SYPL2, TAB1, TBL1XR1, TEAD1, TIMM23, TIMP3, TMEM170A, TMEM260, TMTC1, TP53I11, TRIM4, UBASH3B, UBP1, VAPA, VSTM4, WDR26, ZNF629*
**hsa-miR-320e**	*39326, ABI2, ABR, AGPAT5, AKAP12, ALAD, AMD1, AQP1, ARHGAP19, ARHGEF18, ATP13A3, BAZ1B, BEX1, BLOC1S5, BNIP3L, C16orf72, CACNA1E, CACNB4, CCDC121, CDC25A, CDH11, CEP41, CHD2, CHRNA2, CLASP1, CNOT7, COPA, CPM, CYP26A1, DAB2, DAGLA, DAZL, DCT, DDHD1, DUSP10, DYNC1I1, EIF4EBP2, ENAH, EPHA7, ERP44, FBXO45, GPAM, GPCPD1, GTF2A1, HCN1, HIPK2, IMPAD1, IRS1, JMY, KBTBD6, KCNA6, KLHL28, KLRD1, LAPTM4A, LEPR, LMF1, MAB21L1, MTMR6, NR2C2, NRG1, NTRK2, OCIAD1, ORMDL1, OTUD4, PARP8,* ***PHF6****, PKP4, PLEKHA4, PLEKHA5, PNRC1, POGZ, PPP1R9B, PPP2R5C, PSMA5, RAB33B, RALA, RBM15, RBPJ, RCN2, REPS2, RFX3, RHOG, ROBO2, SAMD4A, SCYL3, SEL1L, SETD5, SKA3, SLC10A3, SLC1A2, SLC22A23, SLC6A17, SORBS2, SOX11, SSSCA1, STK38L, STT3B, TCEA1, TDG, TEAD1, THAP5, TMED7, TMEM47, TMOD3, TRIM27, TSHZ3, ULK1, USP9X, WDR26, YTHDF3, ZC3H7B, ZCCHC3, ZNF3, ZNF500, ZRANB2*
**Upregulated miRNA**	**Regulated Genes**
**hsa-miR-4466**	*RUNX1, SYDE1, FANCA*
**hsa-miR-877-5p**	*ADAMTSL1, ANKFY1, ATP2B1, CDC40, CDKN1B, COL6A3, CSNK1G3, ELF1, FSD1L, FXR2, KRTAP4-9, SORBS3, TP53INP2, UBN2, YLPM1, ZNF174*
**hsa-miR-2355-3p**	*ACTN1, ADAT2, ADD2, AFF4, AKAP2, ANKUB1, ARHGAP20, ARHGEF28, ARID1A, BAZ2B, BTG2, CACNA1A, CAP1, CAPN14, CCDC107, CCPG1, CDON, CHRFAM7A, CNBD2, CTBP1, CYBRD1, DCP2, EHMT1, EP300, EPHA7, ERC2, ERG, ERMP1, ESRRG, ETV1, FAM20B, FAM81A, FAM83B, FGF11, FJX1, FREM2, FRS2, GABRB2, GOLPH3L, GPD2, HAS3, HECW1, IL6ST, ILF2, IRX1, KDM5A, LARP1, LIN28B, LIPA, LPP, LRRN3, MACC1, MEX3C, MPLKIP, MTRNR2L3, MTRNR2L4, OR2C3, PAFAH1B1, PALM2-AKAP2, PARD6B, PBX2, PHEX, PIF1, PLCB1, PLEKHA3, PPIL3, PPM1E, PRSS35, RAB10, RAB27A, RAP2A, RDX, RHBDL2, RNF111, RNF219, RPS6KB1, SCN8A, SERPINE1, SERTAD4, SERTM1, SHROOM4, SRSF10, SSBP3, STAG2, SYT1, TAB2, TADA2A, TAF4, TENM2, TFAP2D, TICAM1, TMEM169, TMEM178A, TMEM9B, TRIM48, TRIM49, TRIM49C, TRIM49D1, WDFY1, XIAP, YPEL2, ZCCHC14, ZFAND3, ZFP91, ZNF235, ZNF484, ZNF554, ZNF692, ZNF716, ZSWIM4*
**hsa-miR-219a-5p**	*ABCB10, ABHD13, ADCYAP1, AKAP13, AKAP6, ANKRD44, ANKRD52, ARHGAP26, ARMC8, ASH1L, ATG14, BTBD7, CAMK1D, CAPS, CC2D1A, CCDC28A, CCNA2, CD164, CDYL2, CGNL1, CHD7, CLASP1, CLOCK, COL4A3BP, COL9A1, CRLF3, CXXC4, CXXC5, DAZAP1, DCAF10, DCBLD2, DDAH1, DIAPH3, DNAJC6, DNAL1, DOK6, EFNB2, ELMOD2, ELOVL7, ERG, ETV5, EYA1, EYA2, FAM199X, FBXO3, FBXO30, FBXO42, FOXJ3, FURIN, GRAMD1B, GXYLT1, HAS3, INPP5J, ISL1, KBTBD8, KCNA4, KCNH8, KIAA1549, LAPTM4A, LEF1, LGALSL, LPP, LSAMP, MBNL1, MECOM, MEF2D, MFNG, MIER3, MMS19, NDRG4, NR2C2, OCRL, PCDH17, PDE4D, PDGFRA, PIGG, PIGR, PPARGC1A, PRDM16, PRKAA2, RAB35, RASSF3, RBM24, RBMS3, RECK, RIMS1, RNF6, ROR1, RPRD2, S100PBP, SCAI, SDK1, SEMA4G, SKIDA1, SLC16A7, SLC31A1, SLC39A10, SLC41A1, SLK, SNRK, SORCS1, SOX14, SOX6, STRBP, SYT5, TENM2, TGFBR2, THRB, THSD7B, TMEM98, TMX4, TNRC18, TPCN1, TRHDE, TSC22D2, TSPAN2, TTBK1, UBASH3B, UBE2Z, UBE3A, UBR1, WEE1, ZBTB18, ZC3H6, ZEB2, ZNF697, ZNF704*
**hsa-miR-4485-3p**	*CTDSPL2, GALNT14, LACTB2*

**hsa-miR-34a-3p**	*ABHD10, ADSS, ANTXR2, APOBEC4, ARHGEF35, CAB39, CADM1, CERS6, CRIPAK, DDX27, DYRK1A, ECHS1, EHMT1, EIF3C, EIF3CL, G3BP2, GALNT1, GORAB, GPN1, HDAC2, HRH4, HSDL1, JUN, KDM5B, KIDINS220, LRP2, MAP3K4, MIEF1, MRC1, MSR1, NMT2, NSD1, PALLD, PARP16, PAXBP1, PCDH19, PDCD6IP,* ***PHF6****, PRSS12, PTAR1, RAB3IP, RFFL, RMI1, RNF135, RNF44, RNPEP, RSRC1, RUNX1, SCRN3, SCYL2, SH3GLB1, SHCBP1, SLC22A5, SNX4, SUMO1, TACC2, TET1, TMEM176B, TNF, UQCC1, USP37, WDR72, XRN2, YAP1, ZC3H15, ZHX1*
**hsa-miR-378a-5p**	*ACSL6, ACTL6A, AKAP17A, AMIGO1, ANKRD46, ANO3, APOO, APPBP2, ARHGEF39, ARL15, ARPC5, ASXL2, B3GALNT1, BCL11B, BICD2, BNC1, C11orf54, CACNA1E, CAMKK2, CCDC117, CCDC88A, CCNY, CEACAM1, CHFR, CLCF1, CNPY1, CREB1, CTBP2, CYB5R4, DGCR8, DOCK11, EML1, ENPP6, ETS1, FAM53B, FEN1, FGFR1OP2, FLG2, FTSJ1, GABRA5, GALNT6, GALNTL6, GAS7, GRIA4, HDX, HOOK3, HOXB2, IPMK, KBTBD4, KCNH5, KIAA1324L, KPNA1, LATS2, LIMD1, LIMS1, LNX1, LRRTM1, LSM11, LYN, MEOX2, MLEC, MPLKIP, MRPS2, MTCP1, MTDH, NDEL1, NDUFA4, NFAT5, NFYA, NME6, NPC1, NR4A3, OTUD3, PARP8, PCBP2, PDE4D, PELI1, PGR, PIAS4, PLXNA2, PPM1E, PTK2, RAB11FIP2, RAG1, RELN, ROBO2, SAP30L, SECISBP2L, SH2D4B, SHC3, SLC24A2, SLC25A26, SLC33A1, SLC39A6, SLC7A11, SMKR1, SOX12, SOX6, SP100, STK4, TECTB, TMEM154, TMEM251, TMOD2, TNFSF14, TNRC6B, TOM1L2, TOMM40L, TRDMT1, TRERF1, TRIAP1, TRIB2, TSPAN9, TUBGCP4, TXNDC17, UBE3A, UBN2, UBR3, UHRF1BP1, UNC5D, USP15, VANGL1, VANGL2, VGLL3, YEATS2, YTHDF2, ZNF451, ZNF618, ZNF75A, ZNF781, ZNF84, ZZEF1*
**hsa-miR-184**	*EPB41L5, NUS1*
**hsa-miR-23a-5p**	*ABCA1, ATXN1, BARX2, C3orf56, CAMK1D, CCDC97, CCNJL, CHD4, DSC1, ERC2, FAM49A, FOXC1, GIPC3, GOLGA6L1, GOLGA6L6, GPC6, HIRIP3, IGF2, KCNC4, KCNIP1, LIPG, MAPRE1, MTMR4, MYEOV, PPA2, REG1A, SSMEM1, TEC, TMEM127, TMEM140, TMEM217, USH2A, VSIG1, ZMYM3*
**hsa-miR-455-3p**	*ACAN, ACP2, ADCY1, ARMC8, BCL2L12, CARF, CD80, CLVS2, CNOT6, COLEC12, CUL3, DYNLL2, EID1, ELF3, EPHB1, FAM227A, FBXO42, FRMD3, FRYL, FXR1, FZD10, GABARAPL2, GLCCI1, GNL1, GPATCH2L, GRAMD2B, GTPBP1, HNRNPR, HOXC4, HSF1, ID2, KIF5A, KMT2A, KMT2C, LCE6A, MARVELD3, MFAP3L, MINDY1, MSANTD3-TMEFF1, NFIB, NLN, NOL4, NOVA1, NR2F1, OTOR, OTULINL, PDE4DIP,* ***PHF6****, PIK3R1, PNPLA6, POU3F3, PPP2CB, PRKAB2, PRKD3, PSIP1, PTPN9, RMND5A, RTN4, RUSC1, SALL1, SAR1A, SCG3, SEC62, SEMA3G, SLC22A4, SLC25A3, SLC35F1, SSR1, STK17B, STRADB, TBC1D24, TENM3, TENM4, TFRC, TMED7, TMEFF1, TMEM206, TPRG1, TPT1, TTK, UBE2K, UBE2Q2, UHRF1BP1, USP30, VEGFC, WDR26, XCL1, XCL2, XPO1, ZBTB18, ZCCHC10, ZFP36L1, ZNF91*
