# Supplementary material for: Exosomes Released by Corneal Stromal Cells Show Molecular Alterations in Keratoconus Patients and Induce Different Cellular Behavior
Source: Biomedicines. 2022 Sep 21;10(10):2348. doi: 10.3390/biomedicines10102348 (PMC9598276; doi:10.3390/biomedicines10102348)
Supplement: Supplementary file 1 [file biomedicines-10-02348-s001.zip › biomedicines-1778042-supplementary/Supplementary Table S6.pdf]

Supplementary Table S6

Genes that encode PG core proteins, or are related to the GAG chains covalently linked to them, predicted as biological targets of miRNAs whose levels appear altered in the exosomes of keratoconic cells

miRNA	Protein symbol	UniPROT	Description / Name
**Proteoglycans**			
**hsa-miR-23a-5p**	GPC6	Q9Y625	Glypican-6
**hsa-miR-3192-5p**	PRELP	P51888	Prolargin
**hsa-miR-6724-5p**	CD44	P16070	CD44 antigen
**Hyaluronic acid synthesis**			
**hsa-miR-2355-3p**<br>**hsa-miR-219a-5p**	HAS3	O00219	Hyaluronan synthase 3
**Glicosaminoglycan Linker modificator**			
**hsa-miR-2355-3p**	FAM20B	O75063	Glycosaminoglycan xylosylkinase
**Chondroitin/Dermatan sulphate modificators**			
**hsa-miR-3223**	CHST11	Q9NPF2	**Carbohydrate sulfotransferase 11**
**hsa-miR-3224**	CHST15	Q7LFX5	Carbohydrate sulfotransferase 15
