# Supplementary material for: Exosomes Released by Corneal Stromal Cells Show Molecular Alterations in Keratoconus Patients and Induce Different Cellular Behavior
Source: Biomedicines. 2022 Sep 21;10(10):2348. doi: 10.3390/biomedicines10102348 (PMC9598276; doi:10.3390/biomedicines10102348)
Supplement: Supplementary file 1 [file biomedicines-10-02348-s001.zip › biomedicines-1778042-supplementary/Supplementary Table S7.pdf]

Supplementary Table S7

List of putative exosomal biomarkers and exclusion biomarkers detected in corneal stromal exosomes. The detection limit was set at molecules represented by at least 2 peptides.

Protein symbol	Normal[1]	Keratoconus[1]
**Exosomal biomarkers**		
SDCBP	-	-
SLC1A5	0.7	0.74
SLC3A2	0.78	0.67
GNB1	0.35	-
CLTC	0.06	-
CD47	-	-
GNB2	0.46	0.55
ITGB1	0.17	0.1
BSG	0.34	-
B2M	-	0.37
ATP1A1	0.05	0.05
RAP1B	-	-
GNAI3	0.15	0.07
**Exclusion biomarkers**		
HMGB1	-	-
HMGB2	-	-
HMGB3	-	-
NOLC1	-	-
SKP1	-	-
SERBP1	-	-
COX5B	-	-
SLIRP	-	-
PTMA	-	-
MAPRE1	-	-
PDAP1	-	-
EIF4B	-	-
EIF4H	-	-
PGM2	-	-
CANX	-	-
HMGB1	-	-
HMGB2	-	-
HMGB3	-	-

[1] Normalized emPAI
